# Circulating granzyme A is elevated within the dysregulated host response and associates with mortality in abdominal sepsis

**DOI:** 10.3389/fimmu.2026.1763092

**Published:** 2026-05-29

**Authors:** Galadriel Pellejero-Sagastizabal, Patricia Esteban, Santiago Letona-Giménez, Alejandro Andrés-Tovar, José Luis Sierra, Ana Senan-Salinas, Eva María Gálvez, Julián Pardo, Elena Morte-Romea, Maykel Arias, José Ramón Paño-Pardo

**Affiliations:** 1 Division of Infectious Diseases, Hospital Clínico Universitario Lozano Blesa, Zaragoza, Spain; 2 Biomedical Research Centre of Aragón (CIBA), Instituto de Investigación Sanitaria Aragón (IIS Aragón), Zaragoza, Spain; 3 Department of Medicine, Psychiatry and Dermatology, University of Zaragoza, Zaragoza, Spain; 4 Centro de Investigación Biomédica en Red en Enfermedades Infecciosas (CIBERINFEC), Instituto de Salud Carlos III (ISCIII), Madrid, Spain; 5 Servicio Urgencias, Hospital Clínico Universitario Lozano Blesa, Zaragoza, Spain; 6 Instituto de Carboquímica ICB-CSIC., Zaragoza, Spain; 7 Department of Microbiology, Pediatrics, Radiology, Preventive Medicine and Public Health, University of Zaragoza, Zaragoza, Spain; 8 Red Española de Terapias Avanzadas (TERAV), and Consorcio Estatal en Red para el Desarrollo de Medicamentos de Terapias Avanzadas (Certera), Instituto de Salud Carlos III, Madrid, Spain

**Keywords:** abdominal sepsis, granzyme A, immune biomarkers, immune networks, innate immunity, mortality, septic shock, soluble immune mediators

## Abstract

**Introduction:**

Granzyme A (GzmA) has been identified as a key amplifier of sepsis-related inflammation in experimental models without contributing to pathogen clearance. However, its behavior and clinical relevance in human bacterial sepsis remain unknown. We aimed to characterize circulating GzmA in secondary peritonitis and explore its associations with clinical severity and all-cause mortality.

**Methods:**

We conducted a single-center prospective cohort study of 42 adults with secondary peritonitis at Lozano Blesa Clinical Hospital (Zaragoza, Spain), with 31 healthy controls. Peripheral blood was obtained at baseline, 24 h, and 48 h. Serum GzmA concentration and activity, GzmB, cytokines, and endothelial and coagulation markers were measured. Analyses included comparisons across Sepsis-3 severity strata and exploratory prognostic models (logistic regression and receiver operating characteristic curves) evaluating associations between baseline biomarkers and mortality. Sensitivity analyses were performed after excluding patients meeting predefined immunosuppression criteria.

**Results:**

At baseline, GzmA concentrations (2.3-fold, q-value = 5.41×10^−12^) and GzmA enzymatic activity (q-value = 6.75 × 10^-^³) were significantly higher in patients than controls, whereas GzmB did not differ (q-value = 0.332). Across baseline severity categories, VCAM-1 showed the clearest gradient (q-value = 0.017), while GzmA concentrations were numerically highest in septic shock (median 165.4 pg/mL). GzmA remained elevated through 48 h, and non-survivors had higher baseline levels than survivors. Each doubling of baseline GzmA was associated with higher overall in-hospital mortality (OR 2.52; 95% CI 1.11-5.75, p=0.028), with associations persisting after adjustment for age and comorbidity. Sensitivity analyses excluding immunosuppressed patients yielded a clearer association between GzmA and baseline severity, and confirmed its association with mortality.

**Discussion:**

Circulating GzmA, but not GzmB, is markedly upregulated, remains elevated during early follow-up, and is preliminarily associated with mortality-related outcomes. These exploratory findings are consistent with experimental models and support further evaluations of extracellular GzmA to investigate its biological and potential translational relevance in sepsis.

## 1 Introduction

Sepsis is a significant global health concern due to its rising incidence, high morbidity, and mortality (1, 2). From a pathophysiological standpoint, sepsis is a complex syndrome characterized by a systemic, dysregulated and excessive host immune response to microbial pathogens (3, 4). Among the underlying infections leading to sepsis, secondary peritonitis stands out as the leading cause of abdominal sepsis and the second most frequent of sepsis overall, worldwide (5, 6). Despite improved surgical techniques, antimicrobial and supportive measures, secondary peritonitis continues to be associated with high complication and mortality rates (7). Beyond these clinical determinants, accumulating evidence indicates that both local and systemic immune responses are critical drivers of patient outcome (8, 9).

Early risk stratification of patients with peritonitis is essential to identify those at highest risk of progression to sepsis, septic shock, and those with poor outcomes. Current clinical tools and biomarkers such as procalcitonin are useful for detecting infection but provide only limited prognostic value, particularly for predicting severity trajectories and mortality (10). On the other hand, some immune mediators that have shown promise as biomarkers of sepsis have also been targeted as immunomodulatory therapies (11, 12). However, despite encouraging results in preclinical models, these strategies have consistently failed in clinical trials (13, 14).

This translational gap reflects the need for a more precise characterization of the human immune response, with biomarkers that not only indicate inflammation but are also mechanistically linked to disease progression. Granzyme A (GzmA), a major effector of the granule exocytosis pathway (15), has recently been identified as a key modulator of inflammation in preclinical models of sepsis (16–19). Mechanistically, human and mouse GzmA promotes cytokine production via TLR4 activation in macrophages and other immune cells (18–20). In addition, preliminary studies found elevated levels of GzmA in both serum and peritoneal fluid of patients with abdominal sepsis (18), albeit the precise clinical utility of GzmA as a biomarker in sepsis remains to be fully explored. Importantly, GzmA is not a key mediator of host protection against the primary infections that trigger sepsis, nor against secondary infections that may arise from treatment-induced immunosuppression (21, 22). This distinguishes GzmA from other immune targets and places it in an optimal position for therapeutic intervention, as its inhibition is unlikely to compromise antimicrobial defense, an issue that has hindered the success of previous immunomodulatory strategies (11, 23).

Here, we sought to characterize the pathogenic role of GzmA in abdominal sepsis and explore preliminary prognostic associations. We prospectively studied patients with secondary peritonitis across the full clinical severity spectrum, from uncomplicated infection to septic shock, examining associations with disease persistence and overall mortality. Our findings provide initial insights into the potential utility of GzmA not only as a marker of inflammation, but also as an effector protease that may contribute to the amplification of immune responses and tissue injury and therefore with a potential role as therapeutic target in sepsis. These exploratory results justify further investigation of GzmA-related pathways through larger translational studies.

## 2 Methods

### 2.1 Study design and setting

We conducted a single-center, observational, prospective cohort study (nested case–control approach) designed primarily as a mechanistic–exploratory analysis to characterize circulating GzmA within the systemic inflammatory and endothelial response in human secondary peritonitis. The study also incorporated a preliminary prognostic evaluation to determine whether early circulating GzmA levels were associated with disease severity and short-term mortality. In parallel with the longitudinal cohort, we performed a cross-sectional comparison with a group of healthy controls to establish reference distributions and disease-specific immune alterations. The study was carried out at Hospital Clínico Universitario Lozano Blesa (Zaragoza, Spain) between 2019 and 2023, under the coordination of the Aragón Health Research Institute network (IIS Aragón). Patient recruitment occurred between February 2019 and June 2020; laboratory determinations, data curation, and statistical analyses were completed thereafter. A subset of patients enrolled early during the recruitment period contributed to our prior translational publication (18), in which human GzmA data were presented to complement mechanistic findings from a murine abdominal sepsis model. The present work extends that cohort with additional patients and broader clinical endpoints.

### 2.2 Study population and recruitment

Patients aged 18 years or older with a confirmed diagnosis of secondary peritonitis, defined as bacterial peritoneal inflammation resulting from intra-abdominal injury or infection requiring surgical intervention, were eligible for inclusion. Exclusion criteria included: absence of baseline blood samples, pregnancy or breastfeeding, and inability to obtain informed consent.

Patients were identified through daily screening by infectious disease specialists, intensivists, surgeons and microbiologists during the recruitment period between February 2019 and June 2020. Clinical and treatment-related data were obtained through prospective review of electronic medical records, pharmacy prescription software, and the hospital’s Intranet system.

Healthy control serum samples were obtained through the Aragón Biobank. Controls were adult donors without acute infection at the time of donation, selected based on sample availability. Samples were processed and stored according to standardized biobank operating procedures. Controls were not individually matched to cases; age and sex are reported in **Table 1** to contextualize between-group comparisons.

**Table 1:** Baseline characteristics, treatment, and clinical outcomes of the peritonitis cohort, and baseline demographics of healthy controls.

Characteristics	Peritonitis cohort (n=42) N (%)	Healthy controls (n=31) N (%)
Age (years), median (range)	68 (28–94)	48 (20–65)
Sex, Female	22 (52.4)	15 (48.4)
Race, Caucasian	42 (100)	N/A
Coexisting conditions		
Charlson Index Median (IQR)	4.50 (2.75–6.00)	N/A
High Charlson Index (≥5)	21 (50)	N/A
Immunosuppression	7 (16.7)	N/A
Cause of immunosuppresion		
-Chemotherapy	4 (9.5)	N/A
-Corticosteroids	1 (2.4)	N/A
-Other immunosuppressors	2 (4.8)	N/A
Infection characteristics		
Peritonitis etiology		
-Biliary	9 (21.4)	N/A
-Bowel perforation	27 (64.3)	N/A
-Appendicitis	4 (9.5)	N/A
-Inflammatory bowel disease	1 (2.4)	N/A
-Gynecologic	1 (2.4)	N/A
Secondary Bacteremia	4 (9.5)	N/A
Baseline Severity		
SOFA basal score Median (IQR)	2.00 (1.00–7.00)	N/A
Sepsis-3 criteria		
-No sepsis	12 (28.6)	N/A
-Sepsis	19 (45.2)	N/A
-Shock	11 (26.2)	N/A
Treatment		
Source control, complete	32 (76.2)	N/A
Time to Surgery (days), median (IQR)	0.5 (0-2)	N/A
Clinical outcomes		
Severity at follow-up (S2) (n=37)		
-Sepsis	10 (27.0)	N/A
-Shock	13 (35.1)	N/A
Length of Hospital Stay, median (IQR)	17.50 (9.00–29.50)	N/A
ICU stay	31 (73.8)	N/A
Length of ICU Stay, median (IQR)	7.00 (4.00–13.00)	N/A
30-day readmission (n=29)	8 (27.6)	N/A
Time to readmission (days), median (IQR)	6.50 (1.25–26.00)	N/A
Overall mortality	13 (31)	N/A
30-day mortality	10 (23.8)	N/A

Data are presented as count (percentage) for categorical variables, median (Interquartile Range, IQR) for continuous variables and median (range) for age. The denominator for all percentages is the full cohort (n=42) unless otherwise specified. Percentages for ‘Severity at follow-up (S2)’ were calculated from the subgroup of patients alive at the time of follow-up (n=37). The percentage for ‘30-day readmission’ was calculated from the subgroup of patients who were discharged alive (n=29).

ICU, Intensive Care Unit; IQR, Interquartile Range; N/A, not applicable; SOFA, Sequential Organ Failure Assessment, S2, follow-up (48 h).

### 2.3 Clinical variables and outcomes

For patient characteristics, we recorded age, sex, ethnicity, comorbidity (assessed using the Charlson Comorbidity Index (24) and immunosuppression status. Immunosuppression status was defined as the presence of any of the following criteria: neutropenia (absolute neutrophil count <1,500 cells/mm³); uncontrolled HIV infection (CD4 count < 200 cells/mm³); active immunosuppressive therapy, including corticosteroids at doses >10 mg/day of prednisone or equivalent for more than 3 months; use of immunosuppressive drugs; or chemotherapy/radiotherapy within the past 3 months. Secondary bacterial peritonitis was defined as inflammation of the peritoneum resulting from intra-abdominal infection, confirmed during surgical intervention (25). Infection-related variables included the underlying cause of peritonitis, presence of secondary bacteremia, and disease severity. Documented causes of peritonitis included appendicitis, inflammatory bowel disease, biliary pathology (including pancreatitis), diverticulitis, intestinal perforation, and gynecologic conditions (26). Uncomplicated peritonitis was defined as secondary peritonitis without evidence of sepsis or septic shock according to Sepsis-3 criteria (4). Secondary bacteremia was defined as bloodstream infection originating from the primary peritoneal focus.

Systemic severity was assessed using the Sequential Organ Failure Assessment (SOFA) score (27), calculated from its six standard components: respiratory function (PaO_2_/FiO_2_), coagulation (platelet count), liver function (bilirubin), cardiovascular status (mean arterial pressure and need for vasopressors), central nervous system status (Glasgow Coma Scale), and renal function (creatinine). Severity categories (no sepsis, sepsis, septic shock) were assigned according to the Sepsis-3 definitions (4), which rely on SOFA as the core measure of organ dysfunction. Evaluations were performed at baseline (D0) and during early follow-up (S1–24 h and S2–48 h). Baseline was defined as the blood sample obtained at the time of diagnosis of secondary peritonitis. Microbiological variables included blood and peritoneal fluid cultures results. Treatment-related variables included the prescribed antimicrobial regimen, source control (complete or incomplete), defined as surgical drainage or debridement of the infectious focus, and time to its achievement.

Outcome variables included persistent sepsis/shock status at S2 and all-cause mortality. Persistent sepsis/shock was defined as sepsis or septic shock at the S2 timepoint, regardless of the baseline category. Overall mortality was defined as in-hospital all-cause mortality (4). The main prognostic endpoint was 30-day all-cause in-hospital mortality; no deaths occurred between days 28 and 30, making 30-day and 28-day mortality equivalent in this dataset (28).

### 2.4 Sample collection and timepoints

Peripheral blood samples were collected at three predefined timepoints: baseline (D0, at the time of diagnosis of secondary peritonitis), S1 (target 24 hours post-surgery) and S2 (target 48 hours post-surgery). In practice, S1 samples were collected predominantly on postoperative day 1 (88%; median 1 day, range 1–4 days) and S2 samples on postoperative day 2 (median 2 days, IQR 2–4 days; range 2–7 days).

Biomarker measurements included granzymes: GzmA, granzyme B (GzmB), and GzmA enzymatic activity; pro-inflammatory cytokines: TNF-α, IL-6, IL-1β, IL-8, IL-17, IFN-γ; anti-inflammatory mediators: IL-10, IL-1Rα; chemokines: CXCL9, CXCL10, CCL3; endothelial markers: ICAM-1, VCAM-1, E-Selectin, VEGF; coagulation markers: D-dimer, von Willebrand Factor (vWF α2) and the classical infection biomarker procalcitonin. All soluble biomarkers were quantified as serum concentrations in pg/mL, whereas GzmA enzymatic activity was expressed in µmol/min.

### 2.5 Sample processing

Peripheral blood was collected into serum separator tubes and allowed to clot at room temperature (RT) for 30 minutes. Samples were then centrifuged for 10 min at 2500 rpm at RT to separate the serum from the cellular fraction. The serum supernatant was carefully collected, aliquoted, and stored at −80 °C until analysis.

Multiplex serum protein analyses Luminex assay was run according to manufacturer’s instructions in 100 µl of serum, using a custom human cytokine panel (R&D Systems, catalogue no. LXSAHM-18). Supernatants were mixed with beads coated with capture antibodies and incubated on a 96 well filter plate for 2 hours. Beads were washed and incubated with biotin-labelled detection antibodies for 1 hour, followed by a final incubation with streptavidin-PE. Assay plates were measured using a Luminex 200 instrument (ThermoFisher, catalogue no. APX10031). Data acquisition and analysis were performed using xPONENT software. The standard curve for each analyte had a five-parameter R2 value > 0.95 with or without minor fitting using xPONENT software.

Human serum GzmA levels from healthy donors and from patients with secondary peritonitis were measured using a commercial ELISA kit (Human Granzyme A DuoSet ELISA, R&D Systems), following the manufacturer’s instructions.

Serum samples were used to evaluate the activity of GzmA using specific quenching FRET fluorescent substrates (FAM-VANRSAS-DABCYL). Briefly, 40 µl of 100 mM Tris-HCl pH 8.5 (buffers for GzmA) were added to flat bottom, black plates, with 10 µl of the serum samples. 50 µl of GzmA substrate were added and the fluorescence of the plate was read at time zero and 1 h for GzmA using 475 nm excitation and 520 nm emission wavelengths. GzmA activity was calculated based on a calibration curve with known concentrations of carboxyfluorescein.

### 2.6 Sample size and statistical analyses

The sample size was not determined by formal statistical power calculations given the exploratory nature of this pilot study; instead, all eligible patients within the study period were included to maximize representativeness.

Categorical variables are presented as counts and percentages. Continuous variables are expressed as mean ± standard deviation (SD) if normally distributed, or median with interquartile range (IQR) otherwise. Comparisons between groups (peritonitis vs. controls, and across severity strata) were performed using the chi-square or Fisher’s exact test for categorical variables, and the Mann–Whitney *U* test for continuous variables, as appropriate. For comparisons involving more than two groups, Kruskal–Wallis tests were applied, followed by Dunn *post-hoc* tests for pairwise comparisons when appropriate. To account for multiple testing within each family of analyses, p values from the two independent GzmA assays (concentration and activity) were adjusted using the Holm method, whereas p values from the multiplex Luminex biomarker panel were adjusted using the Benjamini–Hochberg (BH) false discovery rate procedure (29). To evaluate longitudinal changes, paired Wilcoxon tests were used when comparing each timepoint with controls, p-values were adjusted using the Holm method. Log_2_-transformed GzmA concentrations and enzymatic activity were additionally assessed using linear mixed-effects models with categorical timepoints and within-subject correlation (30). To explore patterns of co-regulation among immune biomarkers over time, we performed an exploratory and descriptive pairwise correlation analysis in the overall peritonitis cohort at baseline, S1, and S2, using Spearman correlation coefficients with BH adjustment for multiple testing and a correlation threshold of |ρ| > 0.3; only associations with BH-adjusted q-value < 0.05 were retained.

For the exploratory biomarker–outcome analyses, we used univariable and multivariable logistic regression models, reporting odds ratios (OR) with 95% confidence intervals (CI). Continuous biomarker variables were modelled as log_2_-transformed values in all regression analyses. To build the multivariable models while minimizing overfitting given the limited number of events, clinical variables were screened separately and analyzed from a causal-inference perspective (**Supplementary Figure 1**), and each biomarker was adjusted for one confounder at a time. Incomplete source control and SOFA were considered downstream of the inflammatory response (and thus possible mediators/colliders) and were not used as confounders; SOFA-adjusted models were interpreted only in a prognostic (AUROC), not mechanistic, framework. Because overall, in-hospital mortality was recorded over heterogeneous follow-up durations, therefore, we used 30-day all-cause mortality as the standardized and clinically accepted prognostic endpoint (31, 32), with overall mortality retained as a sensitivity analysis. Receiver operating characteristic (ROC) analyses were performed to assess the discriminatory ability of each biomarker (33). AUC and its 95% CI were calculated on raw biomarker data using a 1,000-sample stratified bootstrap. Optimum cut-off values were found via the Youden Index method (34). Finally, sensitivity analyses for the main granzyme markers were performed after excluding patients who met the predefined immunosuppression criteria.

All statistical analyses were performed in Python 3.10 (Python Software Foundation) using a Google Colab environment. Data preprocessing and manipulation were conducted with pandas and NumPy. Hypothesis testing and non-parametric procedures were implemented with SciPy, whereas regression modelling and mixed-effects analyses were fitted using statsmodels. ROC analyses and classification performance metrics were obtained with scikit-learn. Visualizations were generated with matplotlib, seaborn, and Plotly.js (v2.35.2). Additionally, R language version 4.3.0 (2023-04–21 ucrt) -- “Already Tomorrow”, platform x86_64-w64-mingw32/x64 (64-bit) on Windows 10 x64 (build 19043) with RStudio development environment version 2022.12.0 + 353 was used for complementary analyses.

### 2.7 Ethical considerations

The study was approved by the Aragón Research Ethics Committee (CEICA; number PI18/023). Written informed consent was obtained from all participants or their legal representatives. Routine clinical blood draws and intraoperative samples were used to minimize patient discomfort. All samples were processed and stored by the Aragón Biobank according to standard operating procedures.

## 3 Results

### 3.1 Study cohort and clinical characteristics

**Figure 1:**
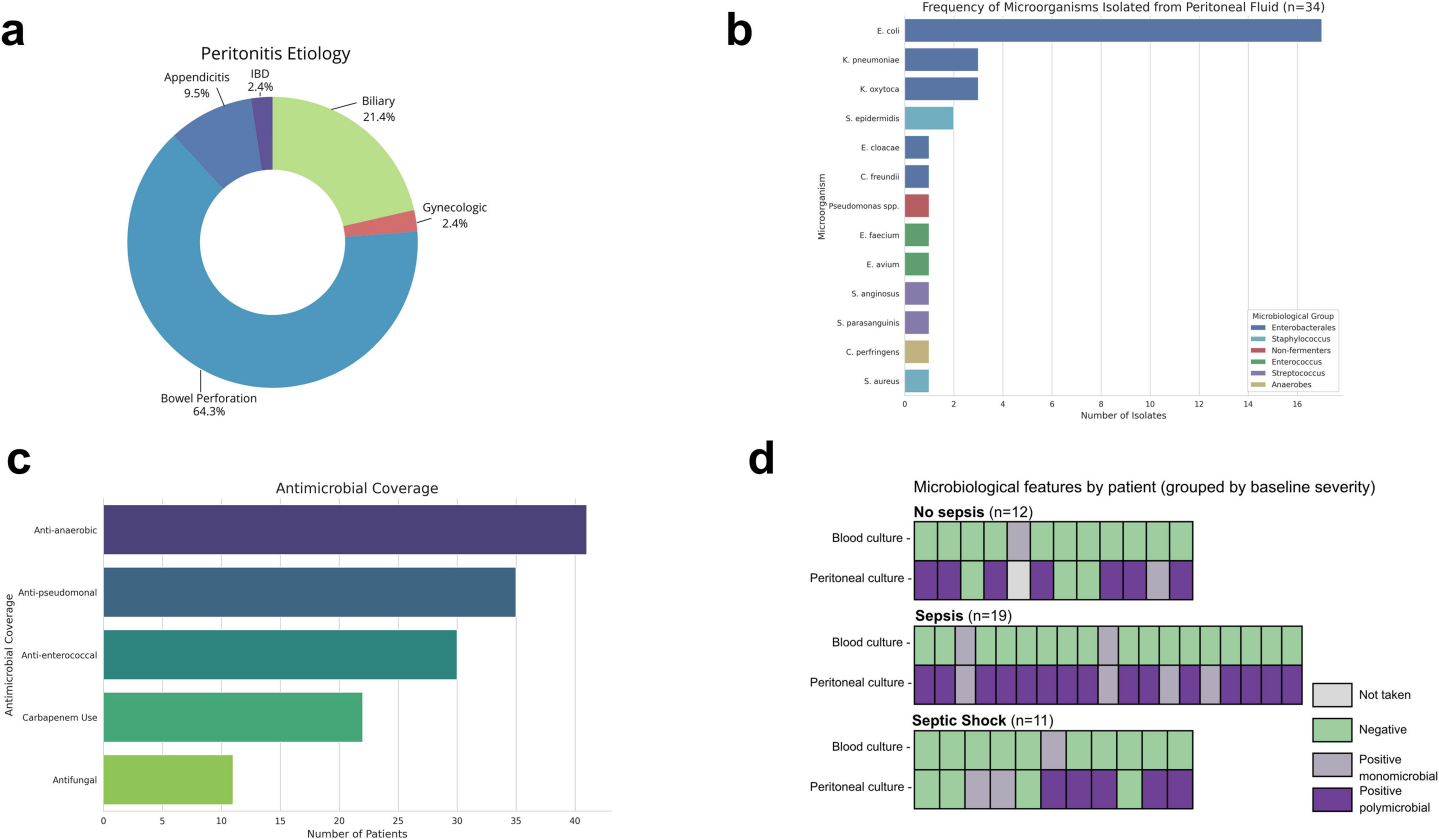
Clinical and microbiological characteristics of the study cohort at baseline. **(A)** Distribution of underlying causes of secondary peritonitis in the study cohort (n=42). **(B)** Frequency of different microorganisms isolated from peritoneal fluid cultures (n=34) at the time of surgical intervention. **(C)** Summary of empirical antimicrobial therapy administered at baseline, classified by spectrum of coverage. Anti-anaerobic coverage included metronidazole, amoxicillin/clavulanic acid, piperacillin/tazobactam, or carbapenems; anti-pseudomonal coverage included piperacillin-tazobactam, cefepime, meropenem or imipenem; and anti-enterococcal coverage included amoxicillin/clavulanic acid, piperacillin-tazobactam, vancomycin, linezolid and tigecycline. Carbapenem use included meropenem, imipenem, or ertapenem, while antifungal coverage included fluconazole, anidulafungin or caspofungin. **(D)** Tile plot showing microbiological culture results from blood and peritoneal fluid for each patient, grouped by baseline clinical severity (No Sepsis, n = 12; Sepsis, n = 19; Septic shock, n = 11). D0, basal measure time; IBD, Inflammatory Bowel Disease; S2, follow up (48 h).

A total of 42 patients with secondary peritonitis and 31 healthy controls were included in the study. Samples were obtained from all 42 patients at baseline, from 39 patients (93%) at S1 and from 37 patients (88%) at S2. Some samples at S1 and S2 were missed due to early discharge or technical reasons. Patients were older adults (median age 68 years) with substantial comorbidity (half had Charlson index ≥4.5) and 17% were immunosuppressed. Most peritonitis episodes arose from bowel perforation (64.3%), or biliopancreatic disease (21.4%) (**Figure 1A**). At baseline, around three quarters of patients fulfilled Sepsis-3 criteria for sepsis or septic shock, and complete surgical source control with early intervention was achieved in most cases (median 0.5 days). Concomitant bacteremia was uncommon (9.5%); microbiological findings and antimicrobial regimens are shown in **Figures 1B–D**. Full baseline characteristics and clinical outcomes are summarized in **Table 1**.

### 3.2 GzmA levels and immune profile at baseline

**Figure 2:**
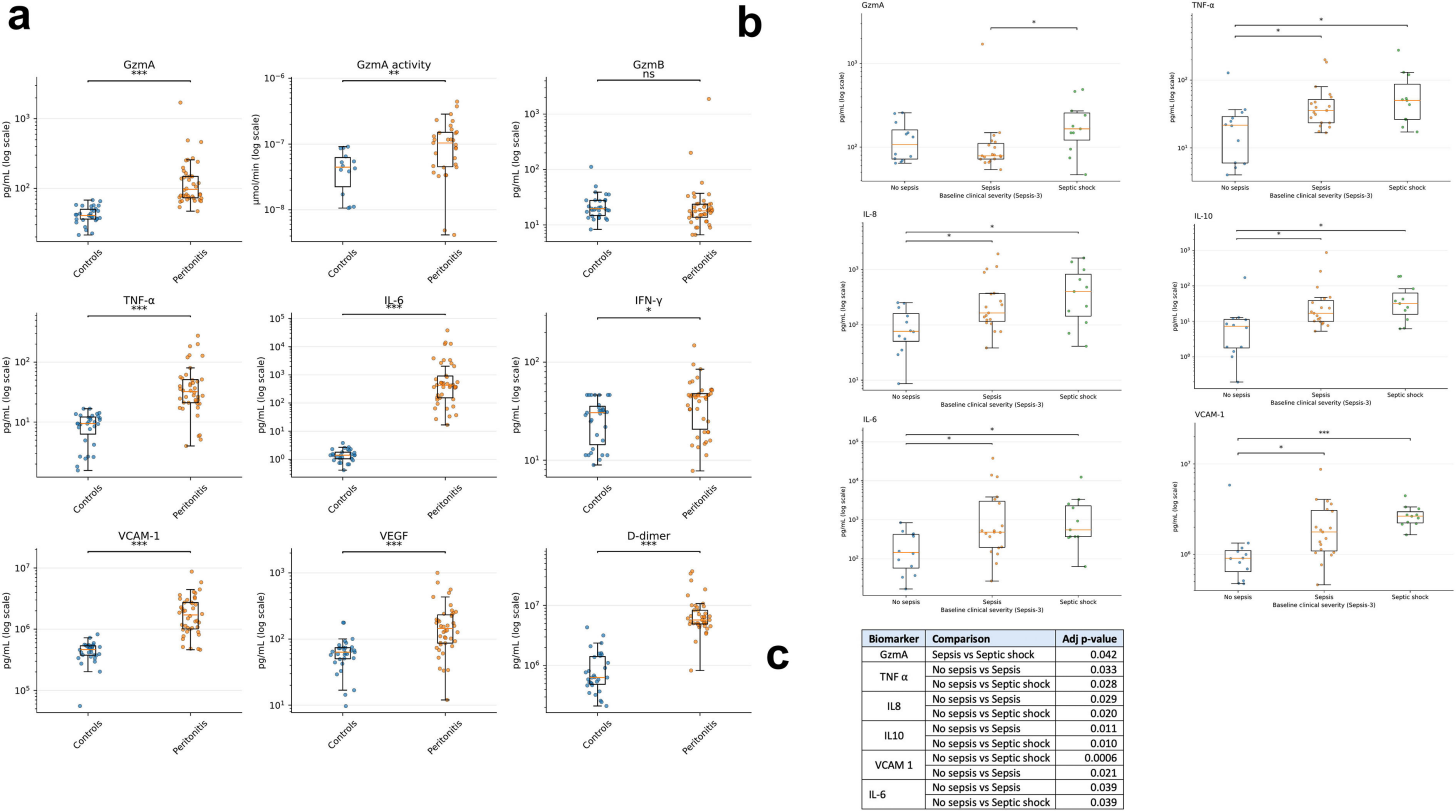
Immune biomarker levels at baseline in patients with peritonitis: comparison with healthy controls and across disease severity. **(A)** Baseline levels of immune biomarkers in patients with peritonitis (n=42) compared to healthy controls (n=31). Boxplots display the median and interquartile range. Comparisons were performed using a two-sided Mann-Whitney U test. Q-values were adjusted using Holm correction for the two GzmA assays and BH correction for the multiplex biomarker panel. All data are plotted on a logarithmic scale. **(B)** Levels of key biomarkers stratified by clinical severity at baseline (No sepsis, n=12; Sepsis, n=19; Septic shock, n=11). Boxplots display the median and interquartile range, with individual patient data overlaid as points. Global differences between the three groups were assessed using the Kruskal-Wallis test. Q-values were adjusted using Holm correction for the two GzmA assays and BH correction for the multiplex biomarker panel. All data are plotted on a logarithmic scale. **(C)** Table of q-values from *post-hoc* pairwise comparisons between severity groups, calculated using Dunn’s test with a BH correction for multiple comparisons. Only statistically significant comparisons are shown. BH, Benjamini-Hochberg; GzmA, granzyme A; GzmB, granzyme B; KW, Kruskal-Wallis test; *p < 0.05, **p < 0.01, ***p < 0.001; ns, not significant.

At the time of surgery, we measured 20 serum markers reflecting immune activation and cardiovascular/endothelial injury. Patients with secondary peritonitis had significantly higher circulating levels of GzmA compared with healthy controls (median 96.96 vs. 40.69 pg/mL; a 2.3-fold increase; q-value = 5.41×10^−12^), as well as greater enzymatic GzmA activity (median 5.50 × 10^-8^ vs 0 [0–4.17 × 10^-8^] µmol/min; q-value = 6.75 × 10^-^³). In contrast, GzmB concentration did not differ between the groups (q-value = 0.332). Other immune mediators as well as markers for vascular damage were significantly increased in patients relative to controls, including IL-6, IL-8, CXCL10, E-Selectin, VCAM-1, and D-dimer (**Figure 2A**; **Supplementary Figure 2**; **Supplementary Table 1**).

When stratified by baseline severity, several inflammatory and endothelial biomarkers showed severity-related gradients (**Figure 2B**; **Supplementary Table 2**). VCAM-1 showed the steepest severity gradient, with an almost threefold increase from no-sepsis to septic shock (q-value 0.017). IL-6, IL-8, IL-10 and TNF-α also showed increasing trends with greater clinical severity. Regarding baseline GzmA, concentrations were highest in patients with septic shock (median 165.4 pg/mL), corresponding to a 1.54-fold increase relative to the no-sepsis group; however, the overall adjusted comparison across severity groups did not remain significant (Kruskal–Wallis raw p = 0.049; q-value = 0.098). In *post-hoc* analysis, GzmA concentrations were significantly higher in septic shock than in sepsis (q-value 0.042; **Figure 2C**). GzmB concentration and GzmA enzymatic activity did not differ between severity groups (**Supplementary Table 2**). Pairwise adjusted *post-hoc* comparisons are detailed in **Figure 2C**.

### 3.3 Longitudinal kinetics of GzmA and immune markers

**Figure 3:**
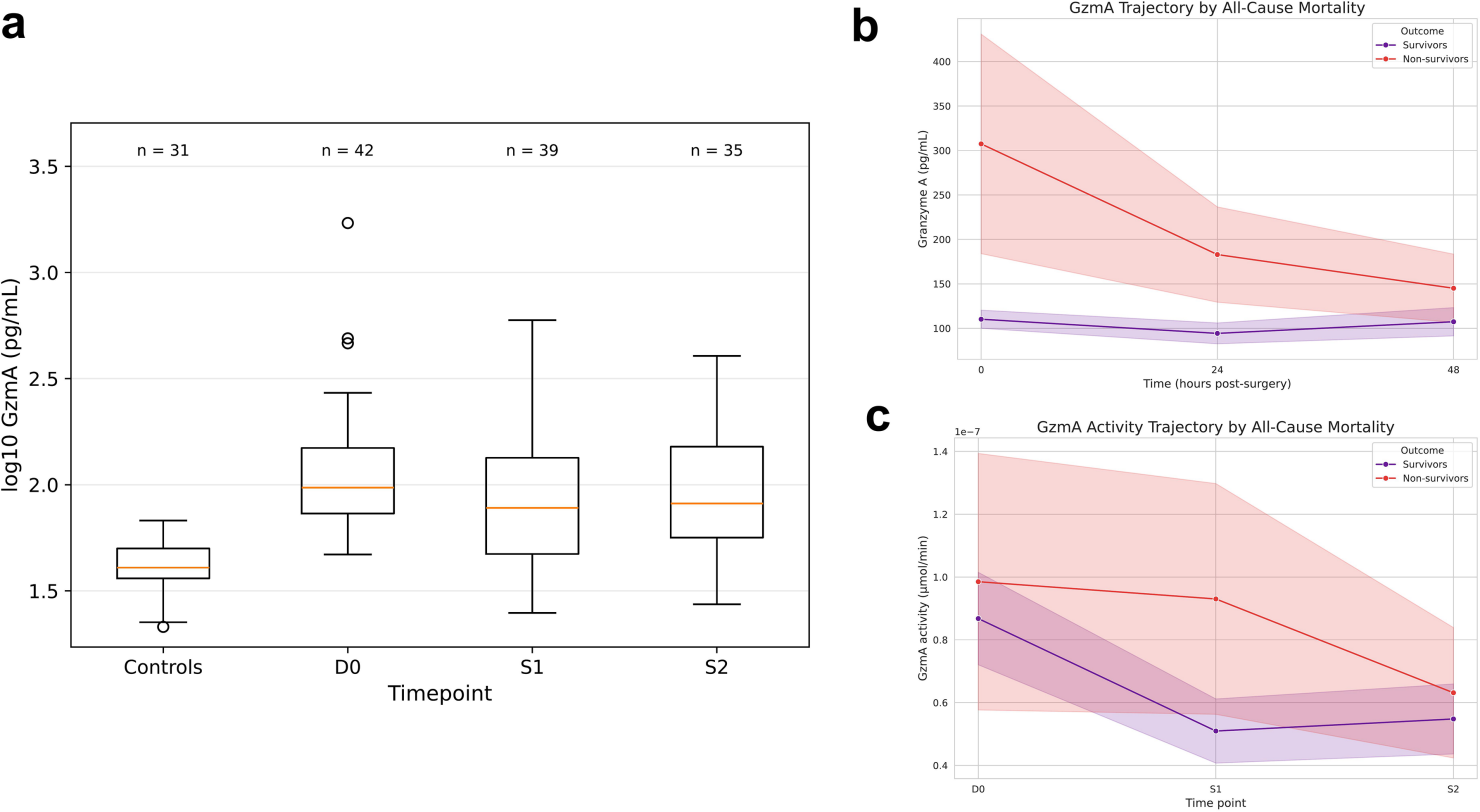
Longitudinal trajectories of circulating granzyme A in secondary peritonitis. **(A)** Boxplots of log10-transformed circulating GzmA concentrations (pg/mL) across healthy controls (n = 31) and the three study timepoints (D0, n=42; S1, n=39; S2, n=35). Boxplots display the median and interquartile range, with whiskers extending to 1.5 × IQR and individual outliers shown as points. **(B)** Median GzmA concentrations over time in survivors and non-survivors, with shaded areas indicating interquartile ranges (IQR). **(C)** Median GzmA enzymatic activity in survivors and non-survivors at each timepoint, with shaded IQRs. D0, baseline (day 0); GzmA, granzyme A; IQR, interquartile range; S1, 24 hours post-surgery; S2, 48 hours post-surgery.

We next examined the temporal evolution of circulating GzmA during follow-up. Overall, GzmA concentrations remained clearly elevated over the first 48 h compared with healthy controls (≈2-fold higher at both S1 and S2; **Supplementary Table 3**; **Figure 3A**). GzmA enzymatic activity also remained elevated relative to controls, but showed little overall change across follow-up within the peritonitis cohort. To assess whether these trajectories differed according to mortality outcome, we modelled log_2_-transformed GzmA concentration and activity using linear mixed-effects models with categorical time (0, 24, 48 h), death status and their interaction (**Supplementary Table 4**). GzmA showed a modest overall early decline but no additional change at 48 h, while non-survivors had consistently higher concentrations at all timepoints (≈1.8-fold higher, p = 0.013), without evidence of divergent kinetics (time×death interaction terms non-significant; **Supplementary Table 4**; **Figure 3B**). In contrast, linear mixed-effects models for GzmA enzymatic activity did not show a clear association with mortality (**Supplementary Table 4**), consistent with the limited separation observed between survivors and non-survivors in **Figure 3C**.

As a complementary exploratory analysis, we examined whether the correlation structure among circulating biomarkers changed over the early course of disease. Correlation heatmaps of the overall cohort at baseline, S1 and S2 are shown in **Supplementary Figure 3**. After filtering for |ρ| > 0.3 and q-value < 0.05, the overall density of significant correlations differed across timepoints. However, a recurrent core involving pro-inflammatory cytokines involving TNF-α, IL-1β, and IFN-γ was retained across baseline, S1, and S2, whereas GzmA-related markers showed limited connectivity under this threshold.

### 3.4 Association of immune biomarkers with clinical outcomes

The previous results provided an overview of the potential involvement of GzmA in the dysregulated immune response during sepsis and shock. To further assess the clinical significance of GzmA, we next analyzed its association, along with other immune modulators, with key clinical patient outcomes.

**Figure 4:**
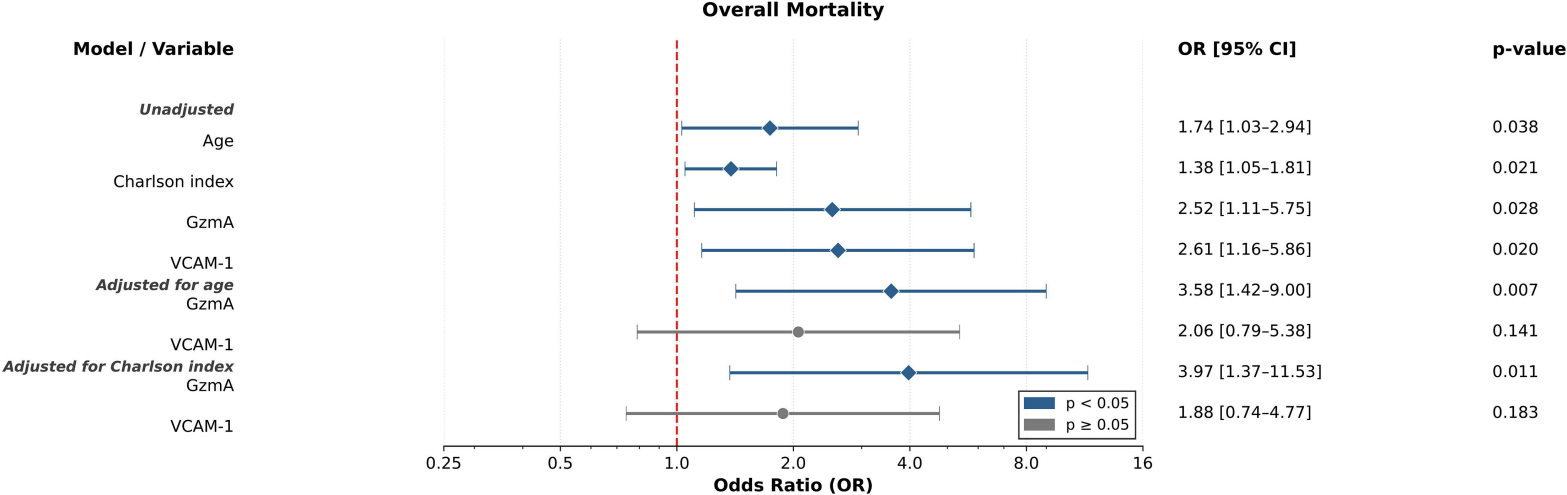
Baseline biomarkers and their association with mortality in secondary peritonitis. Forest plot of baseline biomarkers in relation to overall, in-hospital, all-cause mortality (n=13 events). Squares represent odds ratio (OR) point estimates and horizontal lines indicate 95% confidence intervals (CI); the vertical dashed line represents OR = 1. Biomarker ORs are expressed per doubling of baseline concentration (log_2_-transformed), and clinical predictors per 10-year increase in age and per 1-point increase in Charlson index, as detailed in the Methods. Adjusted models include age and Charlson index. CI, confidence interval; GzmA, granzyme A; OR, odds ratio; S2, follow-up (48 h); SOFA, Sequential Organ Failure Assessment; VCAM-1, vascular cell adhesion molecule-1.

In univariable comparisons (**Supplementary Table 5**), baseline GzmA and VCAM-1 levels were numerically higher in non-survivors than in survivors, with 2.01-fold and 2.14-fold higher median levels, respectively (see **Supplementary Figure 4** for a heatmap-based comparative visualization of these differences across the main clinical contrasts). However, these differences lost significance after adjustment for multiple comparisons (GzmA: raw p = 0.036, q-value = 0.072; VCAM-1: raw p = 0.017, q-value = 0.316). In univariable logistic regression using log_2_-transformed values, each doubling in baseline GzmA and VCAM-1 was associated with higher odds of in-hospital death (OR 2.52; 95% CI 1.11–5.75; p = 0.028 and OR 2.61; 95% CI 1.16–5.86; p = 0.020, respectively) (**Figure 4**; **Supplementary Table 6**). Thus, although the simple group-wise mortality differences were attenuated after multiplicity correction, model-based analyses still supported an association with mortality. To explore potential confounding, pre-specified baseline host factors associated with mortality (age and Charlson index) were evaluated in separate multivariable models. For GzmA, the association with mortality persisted after adjustment for age (OR 3.58; 95% CI 1.42–9.00; p = 0.007) and Charlson index (OR 3.97; 95% CI 1.37–11.53; p = 0.011) (**Figure 4**). For VCAM-1, the mortality association attenuated and lost statistical significance after adjustment for age or comorbidity (**Supplementary Table 6**).

Finally, we assessed predictors of persistent sepsis/shock status at the S2 follow-up, which showed no significant associations for GzmA (**Supplementary Figure 5**; **Supplementary Table 7**).

### 3.5 Discriminatory performance of GzmA and other markers

**Figure 5:**
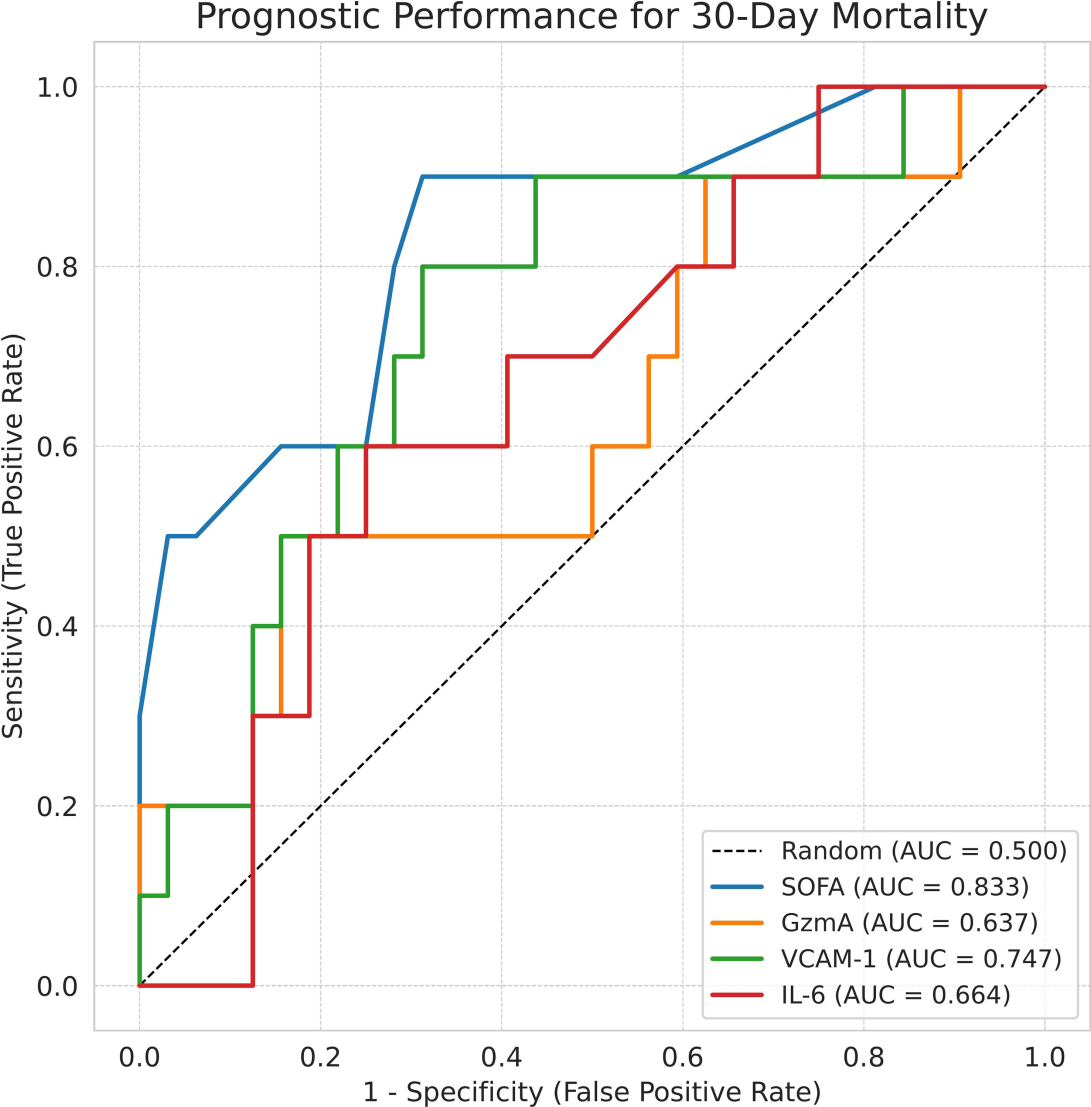
Prognostic value of baseline GzmA and inflammatory biomarkers for 30-day mortality in peritonitis. ROC curves comparing the discriminatory ability of the SOFA score and selected immune biomarkers (VCAM-1, GzmA, IL-6) for 30-day mortality (n=10 events). VCAM-1, GzmA and IL-6 were selected based on their ROC performance and their established mechanistic relevance in sepsis-associated inflammation. AUC values are indicated. The grey dashed line represents the reference line for no discrimination (AUC = 0.5). AUC, area under the curve; GzmA, granzyme A; IL-6, interleukin-6; ROC, receiver operating characteristic; SOFA, Sequential Organ Failure Assessment; VCAM-1, vascular cell adhesion molecule-1.

The associations observed between GzmA levels and mortality prompted us to explore its discriminatory ability for 30-day mortality. In ROC analyses (**Figure 5**), the SOFA score, as expected, showed the highest discriminatory ability for 30-day mortality, whereas GzmA displayed modest discrimination (AUC 0.637; 95% CI 0.422–0.731; p = 0.099). The performance of the full biomarker panel is shown in **Table 2**. In a sensitivity analysis using overall in-hospital mortality, the AUC for GzmA increased to 0.706 (95% CI 0.530–0.881; p = 0.028), indicating statistically significant but still modest discriminatory ability (**Supplementary Table 8**; **Supplementary Figure 6**).

**Table 2:** Discriminatory performance of baseline biomarkers for 30-day mortality.

Biomarker	AUC [CI]	P-value	Cut-off	Sensitivity	Specificity
SOFA	0.833 [0.667-0.958]	0.007	4	0.900	0.688
GzmA	0.637 [0.422-0.834]	0.099	170	0.500	0.844
GzmB	0.503 [0.294-0.731]	0.251	58	0.200	0.969
GzmA Activity	0.341 [0.181-0.528]	0.124	N/A	N/A	N/A
TNF α	0.575 [0.356-0.778]	0.253	200	0.200	1.000
IL6	0.664 [0.478-0.838]	0.241	550	0.600	0.750
IL8	0.650 [0.450-0.828]	0.151	140	0.800	0.500
CXCL10	0.545 [0.344-0.756]	0.825	76	0.500	0.719
IL10	0.591 [0.391-0.775]	0.448	19	0.600	0.625
VEGF	0.534 [0.319-0.734]	0.932	160	0.700	0.594
IL1 β	0.514 [0.300-0.711]	0.652	16	0.600	0.594
IFN-γ	0.573 [0.378-0.777]	0.336	33	0.800	0.406
IL1 Rα	0.603 [0.384-0.797]	0.382	2.1×10^4^	0.600	0.719
CCL3	0.612 [0.412-0.825]	0.679	470	0.800	0.531
Procalcitonin	0.538 [0.341-0.730]	0.544	110	1.000	0.219
IL17	0.463 [0.252-0.680]	0.770	N/A	N/A	N/A
D DIMER	0.614 [0.422-0.809]	0.438	5.1×10^6^	0.800	0.469
E Selectin	0.606 [0.372-0.844]	0.386	8.3×10^4^	0.500	0.906
CXCL9	0.642 [0.444-0.813]	0.128	1300	0.300	0.969
VCAM 1	0.747 [0.575-0.900]	0.026	2×10^6^	0.800	0.688
ICAM 1	0.534 [0.297-0.770]	0.548	1.4 ×10^6^	0.500	0.781

ROC curve analysis was performed in the complete cohort (n = 42 patients with secondary peritonitis; 10 mortality events within 30 days). AUC and its 95% confidence interval were calculated on raw biomarker data using a 1,000-sample stratified bootstrap. Optimal cut-offs were determined using the Youden index. P-values indicate whether the AUC differed significantly from 0.5. N/A is shown for biomarkers with an AUC < 0.50.

AUC, Area under the Curve; CI, Confidence Interval; IQR, Interquartile Range; N/A, Not Applicable; SOFA, Sequential Organ Failure Assessment.

### 3.6 Sensitivity analysis excluding immunosuppressed patients

Finally, given the potential impact of immunosuppression on circulating immune mediators, we performed a sensitivity analysis excluding the 7 patients who met the predefined immunosuppression criteria. Their baseline characteristics and clinical outcomes are shown in **Supplementary Table 9**. Immunosuppression was mainly related to chemotherapy/radiotherapy within the previous 3 months (n = 4), other immunosuppressive drugs (n = 2), or chronic systemic corticosteroids (n = 1).

After exclusion, the immunocompetent cohort included 35 patients. Baseline biomarker analyses were repeated in the restricted cohort. GzmA remained significantly elevated in peritonitis compared with controls (median 105.88 vs 40.69 pg/mL; fold change 2.60; q-value 1.63 × 10^-11^), GzmA activity also remained increased (q-value = 0.006), and GzmB remained non-significant (**Supplementary Table 10**). Across baseline Sepsis-3 strata, GzmA showed a clearer severity-associated gradient, with the highest concentrations in septic shock (median 171.84 pg/mL; q-value = 0.042; septic shock vs. sepsis *post-hoc* q-value = 0.018), while VCAM-1 also remained significantly associated with severity (q-value = 0.046) (**Supplementary Table 11**).

In mortality-stratified comparisons, GzmA was higher in non-survivors than in survivors (median 172 vs. 99.23 pg/mL; p = 0.025; q-value = 0.050) (**Supplementary Table 12**). VCAM-1 and VEGF were also nominally higher in non-survivors, although these differences did not remain significant after multiple-testing correction. In univariable logistic regression, each doubling of baseline GzmA was associated with higher odds of overall in-hospital mortality (OR 2.82; 95% CI 1.10–7.19; p = 0.030), (see **Supplementary Table 13**), with consistent associations after adjustment for age (OR 3.87; 95% CI 1.40–10.69; p = 0.009) and Charlson index (OR 3.50; 95% CI 1.25–9.80; p = 0.017). VCAM-1 was also associated with overall in-hospital mortality in univariable logistic regression (OR 2.86; 95% CI 1.15–7.10; p = 0.023). For persistent sepsis/shock at S2, GzmA was not significantly associated with the outcome (OR 1.83; p = 0.211), whereas IL-8 and procalcitonin were significant predictors and VCAM-1 showed a borderline association (**Supplementary Table 13**). ROC analyses for 30-day mortality, found in **Supplementary Table 14**, showed modest discrimination for GzmA (AUC 0.671; 95% CI 0.436–0.859), whereas GzmA activity and GzmB remained weaker markers. These findings indicate that immunosuppression contributed to biomarker heterogeneity and that its exclusion strengthened several GzmA-related associations.

## 4 Discussion

In this study, we investigated the role of GzmA in secondary peritonitis using two complementary approaches: an exploratory analysis of its biological profile in humans and a preliminary evaluation of its clinical relevance. We found that both serum GzmA concentration and enzymatic activity were markedly increased in patients with peritonitis compared with healthy controls, whereas granzyme B levels did not differ. In parallel, inflammatory and endothelial mediators were elevated, outlining a systemic inflammatory–endothelial activation pattern. Within the peritonitis cohort, GzmA concentrations, but not GzmA activity, were highest in patients with septic shock. Higher baseline GzmA levels were associated with increased overall in-hospital mortality in logistic regression (OR 2.52 per doubling; 95% CI 1.11–5.75), and these associations persisted after adjustment for age and comorbidity, despite only modest overall prognostic discrimination. Importantly, exclusion of immunosuppressed patients reduced biological heterogeneity and strengthened the observed association of GzmA with both baseline clinical severity and mortality. Taken together, these results demonstrate that GzmA, but not GzmB, is elevated in peritonitis, remains persistently increased during early follow-up, and is associated with mortality. To our knowledge, this study provides the first longitudinal characterization of circulating GzmA dynamics in human abdominal sepsis.

GzmA was classically recognized as a cytotoxic serine protease that contributes to target-cell apoptosis through perforin-dependent pathways (35). However, it is now well established that GzmA also exerts important extracellular functions, acting independently of perforin to modulate inflammation (36, 37). In preclinical murine models of bacterial infection, GzmA has proven to be biologically relevant in sepsis by amplifying the inflammatory, coagulation and endothelial response (16, 38, 39). More importantly, experimental work has shown that genetic deficiency or pharmacological inhibition of GzmA markedly reduces systemic cytokine release, endothelial injury, and mortality (18, 20). Murine models have been instrumental in defining these mechanisms, yet translation to human disease remains limited. Building on this analogy, we used a clinical cohort of secondary peritonitis that reproduces key features of the cecal ligation and puncture (CLP) paradigm (17, 40): a polymicrobial intra-abdominal focus, with endogenous flora dominated by *Escherichia coli* (17/34 peritoneal fluid cultures), a full spectrum of clinical severity ranging from localized infection to septic shock, and a standardized management approach including timely surgical source control (median time to surgery 12h).

There is still limited information regarding the behavior and clinical significance of circulating serum GzmA in human bacterial infections. The available evidence derives from a few heterogeneous clinical models but points to a consistent pattern of early extracellular release during the acute phase of infection, seemingly independent of the specific bacterial pathogen (41, 42). In our cohort we corroborated this pattern, observing an early systemic increase in circulating active GzmA across diverse bacterial etiologies, as 79% of cases involved polymicrobial infections combining Gram-positive, Gram-negative, and anaerobic organisms without any major contribution of a specific pathogen.

Moreover, we observed that baseline GzmA concentrations were highest in patients with septic shock, although the overall adjusted comparison across Sepsis-3 categories did not remain significant after correction for multiple testing. This is consistent with previous observations in meningococcal disease, where elevated GzmA levels were detected only in children presenting with shock (43). Similarly, a study of sepsis in the emergency-department reported higher intracellular GzmA in cytotoxic T cells from patients with severe sepsis, with concentrations showing an association with disease severity as measured by the APACHE II score (44).

Data from Gram-negative infections support the concept that GzmA elevation reflects the systemic magnitude of disease severity. In patients with melioidosis, soluble GzmA was higher in patients with bacteremia than in those with localized infection (45). In our cohort, only four patients (9.5%) had documented bacteremia, which is consistent with the low yield of blood cultures in complicated intra-abdominal infection (typically <10%) (46), yet most exhibited clear evidence of immune and endothelial activation. This contradicts the idea that circulating bacteria are required to trigger GzmA release and fits the notion that peritonitis can elicit a disseminated host response that relates to outcomes (47).

In scrub typhus, circulating GzmA levels fall rapidly after effective antimicrobial therapy, reflecting the resolution of an acute, primarily intracellular infection (48). In contrast, in melioidosis, serum GzmA remains elevated for at least 72 hours, indicating sustained systemic immune activation (45). This pattern aligns with our previous translational work in abdominal sepsis (n=10), where GzmA concentrations remained persistently elevated over the first 72 hours compared with healthy donors (18). In the present cohort, circulating GzmA also remained well above control values across follow-up.

In both melioidosis and abdominal sepsis, GzmA elevation occurred in parallel with increases in proinflammatory cytokines, suggesting coordinated activation of cytotoxic and innate immune pathways. Experimental work demonstrates that extracellular GzmA can synergistically enhance TLR-driven cytokine production, particularly IL-6, TNF-α and IL-8, through activation of monocytes and macrophages (16, 37, 49). In our cohort, this cytokine-rich inflammatory profile was evident: IL-6 showed the most pronounced rise compared with controls, followed by IL-8, and was accompanied by endothelial and coagulation activation. As a complementary exploratory approach, we also examined correlation patterns among biomarkers over time to contextualize GzmA within the broader inflammatory–endothelial response. Although these analyses did not support a robust characterization of GzmA-centered correlation structures, data from respiratory infections, where combined models of exhaled volatile organic compounds and serum biomarkers identified IL-6, IL-8, CXCL10, VCAM-1 and GzmA as key predictors of severity in both COVID-19 and non-COVID-19 pneumonia (50), support the existence of a shared dysregulated immune–vascular axis in severe infection rather than a syndrome-specific signature.

The endothelial and coagulation abnormalities observed in our study can also be explained by well-recognized inflammatory pathways. Cytokines activate endothelial NF-κB signaling, which induces VCAM-1, ICAM-1 and E-selectin expression and promotes endothelial dysfunction and activation (51), which in turn activates the coagulation cascade culminating in microvascular thrombosis and elevated D-dimer, consistent with the pattern we observed (52). Although a direct effect of GzmA on endothelial or coagulation proteins has not been demonstrated yet, other closely-related granzymes, like GzmK, can modulate endothelial function through PAR-1 activation (53). We hypothesize that GzmA participates in a broader inflammatory–endothelial–coagulatory network, likely in a context-dependent and non-linear manner, a notion that warrants further mechanistic investigation.

In our cohort, higher baseline GzmA levels were associated with mortality. In logistic regression, increased GzmA was linked to higher overall in-hospital mortality, and this association persisted after adjustment. Although the overall prognostic discrimination of GzmA for 30-day mortality was modest, these findings indicate a graded increase in risk across the GzmA distribution rather than a simple binary separation of survivors and non-survivors. This aligns more closely with trajectory-based biomarkers such as plasma IL-6, IL-8 or angiopoietin-2 (54–56), rather than categorical classifiers like procalcitonin or lactate (57, 58). In this sense, GzmA behaves less as a stand-alone diagnostic classifier and more as a marker of underlying dysregulated host response associated with clinical deterioration. To date, only one prior study, among hospitalized febrile patients with mixed infections, has shown that GzmA independently predicted higher 28-day mortality, even after accounting for shock (59).

In contrast to GzmA, GzmB did not show a clear prognostic signal in our cohort. GzmB is the most potent cytotoxic granzyme and rises in severe infections (60), and prior studies in broader ICU populations have reported stronger associations with mortality (61, 62). However, these studies often included patients with sepsis of heterogeneous origins, making it difficult to extrapolate findings to individual patients, and lacked comparisons with healthy controls, which further complicates the interpretation of GzmB levels. In our homogeneous cohort of abdominal sepsis, GzmB levels did not differ from healthy individuals, even in severe cases, suggesting limited value as a biomarker of disease severity. Nevertheless, the absence of a strong prognostic signal may reflect contextual or temporal factors rather than a lack of biological relevance and supports the notion that GzmA and GzmB operate within distinct immunological axes in sepsis.

Our sensitivity analysis excluding immunosuppressed patients provides a methodological insight for future biomarker studies in sepsis. Previous clinical studies evaluating circulating granzymes in severe infection have included patients with immunomodulating comorbidities, such as malignancy, solid organ transplantation or autoimmune disease, but did not specifically analyze whether immunosuppression modified the observed granzyme–outcome associations (59, 62). In our cohort, the immunosuppressed subgroup was small and did not include patients with profound immunosuppression such as neutropenia, uncontrolled HIV infection or transplantation. Nevertheless, exclusion of these patients strengthened the observed association of GzmA with both baseline clinical severity and mortality. This suggests that baseline immune status may contribute to biomarker heterogeneity, even in relatively moderate forms of immunosuppression. Future studies evaluating GzmA as a biomarker or therapeutic axis in sepsis should therefore prespecify how immunosuppressed populations are handled, either by excluding them from primary biomarker analyses or by analyzing them in immune-status-defined strata.

The possibility that GzmA acts not only as a prognostic marker but as a modifiable amplifier of the host response is supported by multiple murine sepsis models, where GzmA inhibition consistently improves survival, by 30–80%, without impairing bacterial clearance (18, 20). These preclinical findings suggest that GzmA contributes to pathological inflammation rather than microbial control. An important translational caveat is that murine models do not fully reproduce the human cytotoxic granule repertoire. In particular, unlike humans, mice do not express granulysin, a cytotoxic granule protein with antimicrobial activity that can mediate delivery of granzymes into intracellular bacteria (63, 64). Granulysin was not measured in the present study; therefore, we cannot determine whether circulating GzmA reflects a distinct inflammatory pathway or forms part of a broader human cytotoxic-cell activation program involving granulysin. Despite encouraging preclinical data, GzmA inhibitors are not yet available for human use. A key challenge is that the inhibitor used in mice—*serpinb6b*, a natural serpin—is species-specific and does not inhibit human GzmA (65, 66). Alternatives such as antithrombin III, a broad-spectrum serpin used clinically to modulate coagulation, have shown the ability to inactivate human GzmA *in vitro* and suppress GzmA-induced IL-6 release by monocytes, though no clear clinical benefit attributable to this mechanism has been demonstrated (67). In addition, biochemical studies have reported that α2-macroglobulin can bind and inhibit extracellular GzmA, potentially modulating measurable circulating activity (68). Nevertheless, GzmA represents a distinct and promising therapeutic target, unlike prior immunomodulatory approaches that failed to improve outcomes. Anti-cytokine therapies targeting TNF-α, IL-1, or IL-6 broadly suppressed inflammation and often impaired protective immunity (69–72), In contrast, extracellular GzmA blockade may offer a more selective strategy, neutralizing inflammatory amplification without interfering with perforin-mediated intracellular cytotoxicity. This could allow modulation of the immune response while preserving key antimicrobial mechanisms. Future studies should explore whether targeted inhibition of GzmA can safely rebalance the host response in human sepsis without tipping into immunosuppression.

### 4.1 Limitations

This study has several limitations. Its single-center design and modest sample size, particularly within severity and outcome subgroups, limits statistical power and increases the risk of both type I and type II errors. The observational design restricts mechanistic interpretation. In addition, no formal *a priori* sample size calculation was performed given the exploratory, hypothesis-generating aim of the study; effect-size estimates from the earlier pilot dataset were considered insufficiently precise to support reliable power targets for the clinical endpoints and biomarker analyses evaluated here. Accordingly, analysis should be considered exploratory and hypothesis-generating. Although the cohort spans the full Sepsis-3 severity spectrum, it was restricted to a single infection source (secondary peritonitis), which limits generalizability across heterogeneous sepsis syndromes. While longitudinal sampling captured the early in-hospital phase (baseline to 48 h, with variable follow-up up to day 7), it does not encompass the later immunosuppressive trajectory observed in prolonged critical illness. Although we used validated multiplex assays for GzmA concentration and activity, these methods cannot define the cellular source of GzmA or its true bioactivity *in vivo*, which is shaped by local inhibitors, redox state, and compartmentalization. The analytical models were adjusted for age and Charlson index but may still be subject to residual confounding from unmeasured variables. Multivariable and biomarker-combination models may be affected by overfitting given the small number of events. Finally, ROC-derived cutoffs are exploratory and require validation in larger cohorts.

### 4.2 Future perspectives

Our findings identify GzmA as a potential biomarker of early inflammatory and endothelial dysregulation in abdominal sepsis. Although the translational implications remain preliminary, the observed associations between elevated GzmA levels, systemic inflammation, and adverse clinical outcomes supports further investigation of this pathway in hyperinflammatory peritonitis. As sepsis is increasingly recognized as a syndrome of immunological heterogeneity, future work should determine whether extracellular GzmA may help refine patient stratification or inform immunomodulatory strategies in selected subsets of patients as defined by clinical phenotype or molecular profiling (73, 74). Future translational work should aim to validate this therapeutic axis, refine patient selection strategies, including consideration of excluding immunosuppressed populations from primary biomarker analyses, and assess whether modulation of extracellular GzmA can be safely and effectively integrated into precision immunomodulation in sepsis.

## 5 Conclusions

In secondary peritonitis, circulating GzmA is increased at presentation and remains elevated during early follow-up, with the highest levels in patients with septic shock. GzmA levels were observed alongside a cytokine-rich inflammatory profile and markers of endothelial/coagulation activation. This pattern was less evident for GzmB, supporting granzyme-specific associations. Higher GzmA levels were preliminarily associated with mortality in this cohort. Overall, these findings support GzmA as a candidate biomarker linked to the dysregulated host response in abdominal sepsis and provide a rationale for larger, confirmatory studies to evaluate its prognostic utility and to further investigate its biological and potential translational relevance.

## Data Availability

The raw data supporting the conclusions of this article will be made available by the authors, without undue reservation.

## Glossary

APACHE II: Acute Physiology and Chronic Health Evaluation II
AUC: area under the curve
AUROC: area under the receiver operating characteristic curve
CCL3: C-C motif chemokine ligand 3
CD4: CD4-positive T helper lymphocytes
CI: confidence interval
CLP: cecal ligation and puncture
CXCL9: C-X-C motif chemokine ligand 9
CXCL10: C-X-C motif chemokine ligand 10
DAG: Directed acyclic graph
D0: baseline (day 0)
ELISA: enzyme-linked immunosorbent assay
FRET: Förster resonance energy transfer
GzmA: granzyme A
GzmB: granzyme B
HIV: human immunodeficiency virus
HR: hazard ratio
ICAM-1: intercellular adhesion molecule-1
ICU: intensive care unit
IFN-γ: interferon-gamma
IL-1β: interleukin-1 beta
IL-1Rα: interleukin-1 receptor antagonist
IL-6: interleukin-6
IL-8: interleukin-8
IL-10: interleukin-10
IL-17: interleukin-17
IQR: interquartile range
NF-κB: nuclear factor kappa B
NK: natural killer
OR: odds ratio
PAI-1: plasminogen activator inhibitor-1
PaO₂/FiO₂: arterial oxygen partial pressure to inspired oxygen fraction ratio
PAR-1: protease-activated receptor-1
PCT: procalcitonin
ROC: receiver operating characteristic
RT: room temperature
S1: first follow-up timepoint (around 24 h post-surgery)
S2: second follow-up timepoint (around 48 h post-surgery)
SD: standard deviation
Sepsis-3: Third International Consensus Definitions for Sepsis and Septic Shock
SOFA: Sequential Organ Failure Assessment
TLR4: Toll-like receptor 4
TNF-α: tumor necrosis factor alpha
VCAM-1: vascular cell adhesion molecule-1
VEGF: vascular endothelial growth factor
vWF: von Willebrand factor.
