# Supplementary material for: Circulating granzyme A is elevated within the dysregulated host response and associates with mortality in abdominal sepsis
Source: Front Immunol. 2026 May 29;17:1763092. doi: 10.3389/fimmu.2026.1763092 (PMC13259760; doi:10.3389/fimmu.2026.1763092)
Supplement: Supplementary file 1 [file Table1.docx]

Supplementary Material

# Supplementary Data Index

1. Table S1. Comparison of immune biomarker levels in peritonitis patients with healthy controls at baseline.
2. Table S2. Comparison of immune biomarker levels in peritonitis patients at baseline stratified by disease severity.
3. Table S3. Longitudinal dynamics of GzmA concentration and enzymatic activity
4. Table S4. Linear mixed-effects models for longitudinal trajectories of GzmA concentration and enzymatic activity.
5. Table S5. Comparison of immune biomarker levels in peritonitis patients at baseline stratified by overall mortality.
6. Table S6. Baseline biomarker predictors of overall in-hospital mortality: univariable and multivariable logistic regression models.
7. Table S7. Predictors of lack of early clinical improvement at S2 (n=37): univariable and multivariable logistic regression.
8. Table S8. ROC curve sensitivity analysis of baseline immune biomarkers and the SOFA score for predicting overall mortality (n=42, 13 events) in patients with secondary peritonitis.
9. Table S9. Baseline characteristics and clinical outcomes of immunosuppressed patients excluded from the immunocompetent sensitivity analysis
10. Table S10. Baseline immune biomarker levels in immunocompetent peritonitis patients versus healthy controls.
11. Table S11. Baseline immune biomarker levels stratified by baseline disease severity and selected post-hoc pairwise severity comparisons in immunocompetent peritonitis patients (n=35)
12. Table S12. Comparison of immune biomarker levels in immunocompetent peritonitis patients (n=35) at baseline stratified by overall mortality
13. Table S13. Association of biomarkers with outcomes after exclusion of immunosuppressed patients: univariable logistic regression models.
14. Table S14. Discriminatory performance of biomarkers for 30-day in-hospital mortality after exclusion of immunosuppressed patients (n=35, 9 events)
15. Figure S1. Directed acyclic graph (DAG) of hypothesized relationships between GzmA, clinical factors, and mortality in secondary peritonitis.
16. Figure S2. Baseline levels of additional multiplex biomarkers in patients with peritonitis compared with healthy controls.
17. Figure S3. Exploratory correlation heatmaps of immune biomarkers in the overall peritonitis cohort over time.
18. Figure S4. Heatmap of signed fold-change values across key clinical comparisons in secondary peritonitis.
19. Figure S5. Baseline biomarkers and their association with persistent sepsis/shock at S2 in secondary peritonitis.
20. Figure S6. Prognostic performance and survival analyses for overall mortality.

| **Biomarker**  Median [IQR] | **Controls**  (n=31) | **Peritonitis cohort**  (n=42) | **Fold**  **change** | **p-value**  (raw) | **q-value** |
| --- | --- | --- | --- | --- | --- |
| GzmA | 40.69 [33.96–47.42] | 96.96 [73.23–148.98] | 2.38 | 2.71×10^−12^ | 5.41×10^−12^ |
| GzmA activity | 0.00 [0.00– 4.17 × 10⁻⁸] | 5.50 × 10⁻⁸ [1.04×10⁻⁹–1.28×10⁻⁷] | - | 6.75 × 10⁻³ | 6.75 × 10⁻³ |
| GzmB | 19.55 [13.44–25.66] | 17.90 [13.72–23.65] | 0.92 | 0.312 | 0.332 |
| TNF-α | 9.45 [6.48–12.42] | 32.30 [21.08–51.10] | 3.42 | 9.27×10^−10^ | 2×10^−9^ |
| IL-6 | 1.41 [1.01–1.81] | 427.49 [152.88–909.19] | 303.18 | 1.09×10^−12^ | 6.8×10^−12^ |
| IL-8 | 7.76 [5.35–10.17] | 156.20 [76.37–371.77] | 20.13 | 1.04×10^−12^ | 6.8×10^−12^ |
| IL-1β | 5.60 [3.94–7.26] | 14.28 [11.03–20.08] | 2.55 | 4.6×10^−8^ | 8.7×10^−8^ |
| IFN-γ | 30.48 [15.87–45.09] | 44.22 [20.69–47.65] | 1.45 | 0.013 | 0.015 |
| IL-10 | 3.82 [1.89–5.75] | 12.59 [7.98–36.58] | 3.30 | 6.16×10^−8^ | 1.1×10^−7^ |
| IL-1Rα | 467.13 [339.31–594.95] | 7,130 [4,130–29,430] | 15.26 | 4.2×10^−13^ | 6.8×10^−12^ |
| CXCL10 | 23.57 [20.50–26.64] | 56.67 [30.53–111.21] | 2.40 | 3.38×10^−5^ | 4.6×10^−5^ |
| CXCL9 | 557.24 [557.24–557.24] | 557.24 [557.24–718.80] | 1.00 | 0.026 | 0.029 |
| CCL3 | 327.03 [241.45–412.61] | 484.28 [366.21–636.70] | 1.48 | 3.06 × 10⁻⁴ | 3.9×10^−4^ |
| IL-17 | 12.88 [12.88–12.88] | 12.88 [7.37–12.88] | 1.00 | 0.826 | 0.826 |
| D-dimer | 6.31 × 10⁵ [3.51×10⁵–9.11×10⁵] | 5.72 × 10⁶ [4.93×10⁶–8.30×10⁶] | 9.06 | 1.42×10^−12^ | 6.8×10^−12^ |
| VCAM-1 | 4.66 × 10⁵ [3.80×10⁵–5.51×10⁵] | 1.71 × 10⁶ [1.01×10⁶–2.73×10⁶] | 3.67 | 4.74×10^−11^ | 1.3×10^−10^ |
| ICAM-1 | 5.18 × 10⁵ [3.36×10⁵–6.99×10⁵] | 883.94 × 10³ [817.54×10^3^]–1.43×10⁶] | 1.71 | 1.06×10^−5^ | 1.5×10^−5^ |
| E-selectin | 2.02 × 10⁴ [1.53×10⁴–2.52×10⁴] | 5.07 × 10⁴ [3.38×10⁴–7.87×10⁴] | 2.51 | 1.33×10^−10^ | 3.2×10^−10^ |
| vWF-α2 | 4,710 [3,210–6,200] | 16,100 [9,160–27,200] | 3.42 | 6.96×10^−12^ | 2.6×10^−11^ |
| Procalcitonin | 38.41 [23.45–53.37] | 988.45 [298.35–1,650.00] | 25.73 | 1.49×10^−11^ | 4.7×10^−11^ |
| VEGF | 63.22 [51.29–75.15] | 144.19 [86.68–232.53] | 2.28 | 1.47×10^−6^ | 2.3×10^−6^ |

**Table S1**. **Comparison of baseline immune biomarker levels in peritonitis patients with healthy controls.**

Comparisons between peritonitis patients and healthy controls were performed using two-sided Mann–Whitney U tests. Fold change was calculated as the ratio of the median value in the peritonitis cohort to the median value in healthy controls. q-values were calculated using the Holm method for GzmA concentration and GzmA activity, and the Benjamini–Hochberg false discovery rate procedure for biomarkers measured in the multiplex Luminex panel.

Abbreviations: IQR, interquartile range.

**Table S2. Comparison of immune biomarker levels in peritonitis patients at baseline stratified by disease severity.**

| **Biomarker** Median [IQR] | **No-sepsis**  **(n=12)** | **Sepsis**  **(n=19)** | **Shock**  **(n=11)** | **Fold change (Shock/No-sepsis)** | **p-value**  **(raw)** | **q-value** |
| --- | --- | --- | --- | --- | --- | --- |
| GzmA | 107.38 [71.97–160.89] | 78.76 [72.03–110.48] | 165.43 [121.55–255.98] | 1.54 | **0.049** | 0.098 |
| GzmA  activity | 8.68 × 10⁻⁸  [3.41 × 10⁻⁸–1.17 ×10⁻⁷] | 3.38 × 10⁻⁸  [0–1.09 × 10⁻⁷] | 6.11 × 10⁻⁸  [2.55 × 10⁻⁸–1.92 × 10⁻⁷] | 0.70 | 0.328 | 0.632 |
| GzmB | 17.51 [12.96–20.91] | 17.65 [14.87–23.91] | 18.66 [14.20–25.63] | 1.07 | 0.801 | 0.801 |
| TNF ⍺ | 21.59 [5.97–28.96] | 35.64 [23.44–51.83] | 50.11 [26.39–86.93] | 2.32 | **0.019** | 0.094 |
| IL-6 | 145.93 [57.48–422.29] | 480.54 [195.56–2,999] | 552.08 [370.00–2,290] | 3.78 | **0.032** | 0.122 |
| IL-8 | 76.44 [50.45–159.89] | 164.47  [115.66–368.86] | 401.68 [144.19–829.20] | 5.25 | **0.015** | 0.093 |
| IL-1β | 10.92 [9.36–15.56] | 17.84 [12.26–26.32] | 13.14 [12.27–17.58] | 1.20 | 0.067 | 0.130 |
| IFN-γ | 25.57 [14.99–38.74] | 46.21 [36.53–52.38] | 46.21 [37.72–46.21] | 1.81 | 0.062 | 0.130 |
| IL-10 | 7.22 [1.77–11.36] | 16.94 [10.00–38.75] | 31.80 [15.82–62.74] | 4.40 | **0.006** | 0.056 |
| IL-1R⍺ | 6,468  [3,031–1.59 × 10⁴] | 9,250 [5,423–4.4 × 10⁴] | 6,810 [5,300–3.15 × 10⁴] | 1.05 | 0.231 | 0.275 |
| CXCL10 | 43.41 [19.62–66.90] | 52.24 [33.70–79.90] | 177.77 [62.41–207.09] | 4.10 | 0.098 | 0.139 |
| CXCL9 | 557.24  [532.34–572.04] | 557.24  [524.80–611.91] | 873.66 [557.24–1,088] | 1.57 | 0.079 | 0.130 |
| CCL3 | 388.76  [243.36–525.78] | 553.52  [374.48–646.37] | 493.28 [451.28–628.98] | 1.27 | 0.124 | 0.158 |
| IL-17 | 12.88 [7.58–12.88] | 12.88 [9.10–12.88] | 8.62 [6.66–11.96] | 0.67 | 0.254 | 0.275 |
| D-dimer | 5.23 × 10⁶  [4.49 × 10⁶–8.47 × 10⁶] | 6.66 × 10⁶  [5.05 × 10⁶–1 × 10⁷] | 5.05 × 10⁶  [4.78 × 10⁶–6.22 × 10⁶] | 0.97 | 0.102 | 0.139 |
| VCAM-1 | 9.05 × 10⁵  [6.46 × 10⁵–1.1 × 10⁶] | 1.77 × 10⁶  [1.09 × 10⁶–3.07 × 10⁶] | 2.64 × 10⁶  [2.23 × 10⁶–2.96 × 10⁶] | 2.92 | **<0.001** | **0.017** |
| ICAM-1 | 9.67 × 10⁵  [8.24 × 10⁵–1.24 × 10⁶] | 9.67 × 10⁵  [8.18 × 10⁵–1.7 × 10⁶] | 8.18 × 10⁵  [7.61 × 10⁵–9.31 × 10⁵] | 0.85 | 0.261 | 0.275 |
| E-selectin | 4.51 × 10⁴  [2.89 × 10⁴–6.98 × 10⁴] | 5.43 × 10⁴ [4.44 × 10⁴–8.66 × 10⁴] | 4.23 × 10⁴ [3.2 × 10⁴–7.21 × 10⁴] | 0.94 | 0.082 | 0.130 |
| vWF α2 | 1.58 × 10⁴  [1.4 × 10⁴–2.46 × 10⁴] | 2.27 × 10⁴  [1.42 × 10⁴–3.03 × 10⁴] | 9,160  [9,160–2.08 × 10⁴] | 5.80 | 0.081 | 0.130 |
| Procalcitonin | 338.10 [68.45–1,155] | 986.62 [418.92–1,650] | 1,650 [953.67–1,918] | 4.88 | 0.057 | 0.130 |
| VEGF | 98.14 [81.67–115.94] | 153.43 [94.47–219.05] | 223.60 [157.67–351.85] | 2.28 | 0.063 | 0.130 |

Global comparisons across baseline severity groups were performed using the Kruskal–Wallis test. Post-hoc pairwise comparisons were assessed using Dunn’s test. Fold change was calculated as the ratio of group medians (Septic shock/no-sepsis). Q-values for GzmA concentration and GzmA activity were adjusted using the Holm method, whereas q-values for the multiplex Luminex panel were adjusted using the Benjamini–Hochberg false discovery rate procedure. P-values < 0.05 were considered statistically significant and are highlighted in bold.

**Table S3. Longitudinal dynamics of GzmA concentration and enzymatic activity**

| **Biomarker**  **/Timepoint** | **Median** | **IQR (Q1–Q3)** | **Fold-change vs controls** | **p vs D0** | **q-value vs D0** |
| --- | --- | --- | --- | --- | --- |
| **GzmA** | | | | |  |
| D0 | 96.96 | 73.23–148.98 | 2.38 | N/A | N/A |
| S1 | 77.83 | 47.16–133.93 | 1.91 | **0.035** | 0.069 |
| S2 | 81.56 | 56.41–151.21 | 2.00 | 0.334 | 0.334 |
| **GzmA Activity** | | | | |  |
| D0 | 5.50×10^-8^ | 1.04 ×10^-9^–1.28×10^-7^ | N/A | N/A | N/A |
| S1 | 4.79×10^-8^ | 0.00–7.63×10^-8^ | N/A | **0.019** | **0.030** |
| S2 | 5.47×10^-8^ | 8.30×10^-10^ –8.64×10^-8^ | N/A | **0.015** | **0.030** |

^P-values correspond to paired Wilcoxon tests versus baseline, with q-values using the Holm method separately for GzmA concentration and GzmA enzymatic activity across the two follow-up timepoints^**^.^** ^Fold-change values for GzmA concentration are reported relative to healthy controls (median 40.7 pg/mL).^ ^Sample sizes: D0 (n = 42), S1 (n = 39), S2 (n = 35).^

^Abbreviations:^ ^D0, baseline (day 0); GzmA, granzyme A; IQR, interquartile range; N/A, not applicable; S1, early follow-up (24 h); S2, late follow-up (48 h).^

**Table S4. Linear mixed-effects models for longitudinal trajectories of GzmA concentration and enzymatic activity.**

| **Biomarker** | **Effect** | **Estimate (log₂)** | **SE** | **95% CI** | **p-value** |
| --- | --- | --- | --- | --- | --- |
| GzmA concentration | D0 (survivors) | 6.696 | 0.185 | 6.333–7.059 | **<0.001** |
|  | S1 (vs D0, survivors) | -0.284 | 0.136 | -0.550 – -0.018 | **0.037** |
|  | S2 (vs D0, survivors) | -0.159 | 0.142 | -0.437–0.119 | 0.261 |
|  | Non-survivors (overall difference) | 0.825 | 0.333 | 0.172–1.477 | **0.013** |
|  | S1 × non-survivor | -0.037 | 0.245 | -0.518–0.444 | 0.879 |
|  | S2 × non-survivor | -0.227 | 0.255 | -0.726–0.272 | 0.373 |
| GzmA Activity | D0 (survivors) | -24.593 | 0.460 | -25.494–-23.692 | **<0.001** |
|  | S1 (vs D0, survivors) | -0.731 | 0.388 | -1.491–0.029 | 0.060 |
|  | S2 (vs D0, survivors) | -0.487 | 0.405 | -1.280–0.306 | 0.229 |
|  | Non-survivors (overall difference) | -0.891 | 0.826 | -2.511–0.728 | 0.281 |
|  | S1 × non-survivor | 0.993 | 0.700 | -0.380–2.366 | 0.156 |
|  | S2 × non-survivor | 0.490 | 0.725 | -0.931–1.911 | 0.499 |

Models include log₂-transformed biomarker values with categorical time (baseline D0, S1, S2), mortality status, and time–mortality interactions, with a random intercept for each patient. Estimates represent mean differences on the log₂ scale relative to baseline survivors. Negative values indicate decreases compared with baseline; positive values indicate higher levels. Non-survivor effects reflect the overall difference across all time points. Bold p-values indicate statistical significance (p<0.05).

Abbreviations: D0, baseline (day 0); GzmA, granzyme A; log₂, base-2 logarithm; S1, early follow-up (24 h); S2, late follow-up (48 h).

**Table S5. Comparison of immune biomarker levels in peritonitis patients at baseline stratified by overall mortality.**

| **Biomarker/**  **Subgroup** | **Survivors**  **Median** [**IQR**] | **Non-survivors**  **Median** [**IQR**] | **Fold**  **change** | **p-value** | **q-value** |
| --- | --- | --- | --- | --- | --- |
| GzmA | 82.47 [71.79-143.23] | 165.43 [80.59-270.94] | 2.01 | **0.036** | 0.072 |
| GzmA Activity | 7.24×10⁻⁸ [3.26×10⁻⁸-1.31×10⁻⁷] | 3.38 × 10⁻⁸ [0-1.14 × 10⁻⁷] | 0.47 | 0.475 | 0.475 |
| GzmB | 18.55 [15.18-23.26] | 17.65 [11.53-24.03] | 0.95 | 0.615 | 0.891 |
| TNF ⍺ | 31.20 [21.19-53.55] | 33.54 [21.04-47.42] | 1.08 | 0.703 | 0.891 |
| IL6 | 377.73 [135.96-527.16] | 552.08 [349.40-3322.39] | 1.46 | 0.196 | 0.732 |
| vWF-α2 | 1.64 × 10⁴ [9160-2.76×10⁴] | 1.50 × 10⁴ [9160.00-2.33 × 10⁴] | 0.91 | 0.701 | 0.891 |
| IL8 | 125.39 [75.40-252.09] | 217.46 [139.14-401.68] | 1.73 | 0.231 | 0.732 |
| CXCL10 | 57.06 [29.50-91.78] | 52.24 [33.64-117.69] | 0.92 | 0.860 | 1.000 |
| IL10 | 12.11 [7.601-33.98] | 20.37 [10.18-37.44] | 1.68 | 0.399 | 0.758 |
| VEGF | 126.71 [82.86-185.13] | 192.08 [157.14-240.88] | 1.52 | 0.135 | 0.732 |
| IL1 β | 13.14 [10.56-25.56] | 15.43 [12.18-17.78] | 1.17 | 0.913 | 1.000 |
| IFN-γ | 43.70 [19.32-51.52] | 46.21 [33.29-46.21] | 1.06 | 0.702 | 0.891 |
| IL1 R⍺ | 6810.00 [3532.50-2.62 × 10⁴] | 2.12 × 10⁴ [5143.99-5.26 × 10⁴] | 3.11 | 0.301 | 0.734 |
| CCL3 | 464.43 [364.73-635.26] | 524.67 [432.86-640.37] | 1.13 | 0.586 | 0.891 |
| Procalcitonin | 990.28 [151.02-1650] | 887.71 [426.89-1650.00] | 0.90 | 0.978 | 1.000 |
| IL17 | 12.88 [7.689-12.88] | 9.427 [3.449-12.88] | 0.73 | 0.316 | 0.734 |
| D Dimer | 5.41×10⁶ [4.63×10⁶-7.34×10⁶] | 6.43 × 10⁶ [5.06 × 10⁶-1.01 × 10⁷] | 1.19 | 0.145 | 0.732 |
| E Selectin | 4.66×10⁴ [3.29×10⁴-7.25×10⁴] | 5.43 × 10⁴ [3.39 × 10⁴-8.34 × 10⁴] | 1.17 | 0.348 | 0.734 |
| CXCL9 | 557.24 [523.53-703.42] | 557.24 [557.24-938.52] | 1.00 | 0.174 | 0.732 |
| VCAM 1 | 1.18×10⁶ [9.11×10⁵-2.27×10⁶] | 2.52 × 10⁶ [1.96 × 10⁶-3.20 × 10⁶] | 2.14 | **0.017** | 0.316 |
| ICAM 1 | 8.56×10⁵ [8.18×10⁵-1.26×10⁶] | 9.50 × 10⁵ [7.27 × 10⁵-1.46 × 10⁶] | 1.11 | 1.000 | 1.000 |

Comparisons between survivors and non-survivors were performed using two-sided Mann–Whitney U tests. Data are presented as median [IQR]. Fold change was calculated as the ratio of the median value in non-survivors to that in survivors. Q-values were calculated using the Holm method for GzmA concentration and GzmA activity, and the Benjamini–Hochberg false discovery rate procedure for biomarkers measured in the multiplex Luminex panel. Significant p-values (p < 0.05) are shown in bold.

Abbreviations: IQR, interquartile range.

**Table S6. Baseline biomarker predictors of overall in-hospital mortality: univariable and multivariable logistic regression models (n=42).**

| **Model** | **Predictor** | **Odds Ratio** | **95% CI** | **p-value** |
| --- | --- | --- | --- | --- |
| Univariable | log2 (GzmA) | 2.52 | [1.11–5.75] | **0.028** |
|  | log2 (VCAM-1) | 2.61 | [1.16–5.86] | **0.020** |
|  | Age (per decade) | 1.74 | [1.03–2.94] | **0.038** |
|  | Charlson index (per unit) | 1.38 | 1.05 - 1.81 | **0.021** |
| Multivariable  Adjusted for  Age | log2 (GzmA) | 3.58 | [1.42–9.00] | **0.007** |
|  | Age | 2.46 | [1.20–5.04] | **0.014** |
|  | log2 (VCAM-1) | 2.06 | [0.79–5.38] | 0.141 |
|  | Age | 1.34 | [0.71–2.54] | 0.368 |
| Multivariable  Adjusted for Charlson | log2 (GzmA) | 3.97 | [1.37–11.53] | **0.011** |
|  | Charlson Index | 1.64 | [1.14 - 2.37] | **0.008** |
|  | log2 (VCAM-1) | 1.88 | [0.74–4.77] | 0.183 |
|  | Charlson Index | 1.02 | [0.99–1.05] | 0.211 |

P-values < 0.05 were considered statistically significant (bold).

Abbreviations: CI, confidence interval; GzmA, granzyme A; OR, odds ratio; SOFA, Sequential Organ Failure Assessment; VCAM-1, vascular cell adhesion molecule-1.

**Table S7**. **Predictors of persistent sepsis/shock at S2 in secondary peritonitis (n = 37): univariable and multivariable logistic regression.**

| **Variable** | **Univariable OR [95% CI]** | **p-value** | **Multivariable OR [95% CI]**  **(Adjusted for Age)** | **p-value** | **Multivariable OR [95% CI]**  **(Adjusted for Charlson)** | **p-value** |
| --- | --- | --- | --- | --- | --- | --- |
| **Clinical Predictors** | | | | |  |  |
| Charlson Index | 1.53 [1.12 - 2.08] | **0.008** | - | - | - | - |
| Age | 1.28 [0.80–2.06] | 0.302 | - | - | - | - |
| **Biomarker Predictors** | | | | |  |  |
| VCAM-1 | 3.92 [1.60–9.59] | **0.003** | 7.43 [1.73–32.0] | **0.007** | 2.66 [0.94–7.56] | 0.066 |
| IL-8 | 2.63 [1.38–5.01] | **0.003** | 3.14 [1.38–7.15] | **0.006** | 2.51 [1.17–5.35] | **0.017** |
| E-Selectin | 2.27 [0.91–5.66] | 0.078 | - | - |  |  |
| IL-10 | 2.17 [1.25–3.79] | **0.006** | 2.13 [1.14–3.98] | **0.018** | 2.08 [1.07–4.03] | **0.030** |
| TNF-α | 2.41 [1.21–4.82] | **0.013** | 2.31 [1.06–5.02] | **0.035** | 2.48 [1.06–5.82] | **0.036** |
| Procalcitonin | 1.86 [1.22–2.83] | **0.004** | 2.95 [1.42–6.12] | **0.004** | 5.43 [1.50–19.68] | **0.010** |
| CXCL10 | 1.60 [0.99–2.59] | 0.058 | - | - |  |  |
| IL1 R⍺ | 1.29 [0.94–1.79] | 0.117 | - | - |  |  |
| IL-6 | 1.46 [1.06–2.02] | **0.020** | 1.52 [1.05–2.20] | **0.028** | 1.61 [1.06–2.46] | **0.027** |
| IL1 β | 1.48 [0.70–3.12] | 0.300 | - | - |  |  |
| GzmA | 1.29 [0.67–2.48] | 0.443 | - | - |  |  |

P-values < 0.05 are highlighted in **bold**.

Abbreviations: CI, confidence interval; GzmA, granzyme A; OR, odds ratio; SOFA, Sequential Organ Failure Assessment

**Table S8. ROC curve sensitivity analysis of baseline immune biomarkers and the SOFA score for predicting overall mortality (n=42, 13 events) in patients with secondary peritonitis.**

| **Biomarker** | **AUC CI** | **P-value** | **Cut-off** | **Sensitivity** | **Specificity** |
| --- | --- | --- | --- | --- | --- |
| SOFA | 0.845 [0.706-0.951] | **0.002** | 4 | 0.923 | 0.759 |
| GzmA | 0.706 [0.530-0.881] | **0.028** | 170 | 0.538 | 0.897 |
| GzmB | 0.450 [0.255-0.649] | 0.513 | N/A | N/A | N/A |
| GzmA Activity | 0.430 [0.223-0.631] | 0.348 | N/A | N/A | N/A |
| TNF ⍺ | 0.538 [0.353-0.729] | 0.371 | 17 | 1.000 | 0.207 |
| IL6 | 0.627 [0.446-0.798] | 0.208 | 550 | 0.538 | 0.759 |
| IL8 | 0.618 [0.435-0.798] | 0.278 | 140 | 0.769 | 0.517 |
| CXCL10 | 0.519 [0.330-0.706] | 0.893 | 76 | 0.462 | 0.724 |
| IL10 | 0.584 [0.406-0.759] | 0.481 | 19 | 0.615 | 0.655 |
| VEGF | 0.647 [0.446-0.825] | 0.164 | 160 | 0.769 | 0.655 |
| IL1 β | 0.488 [0.321-0.673] | 0.504 | N/A | N/A | N/A |
| IFN-γ | 0.538 [0.374-0.710] | 0.423 | 33 | 0.769 | 0.414 |
| IL1 R⍺ | 0.602 [0.398-0.796] | 0.419 | 2.1×10^4^ | 0.538 | 0.724 |
| CCL3 | 0.554 [0.377-0.743] | 0.976 | 470 | 0.692 | 0.517 |
| Procalcitonin | 0.504 [0.333-0.687] | 0.853 | 100 | 1.000 | 0.207 |
| IL17 | 0.403 [0.228-0.598] | 0.378 | N/A | N/A | N/A |
| D Dimer | 0.643 [0.451-0.801] | 0.321 | 5×10^6^ | 0.846 | 0.483 |
| E Selectin | 0.593 [0.385-0.788] | 0.465 | 8.3×10^4^ | 0.385 | 0.897 |
| CXCL9 | 0.633 [0.459-0.793] | 0.140 | 530 | 1.000 | 0.276 |
| VCAM 1 | 0.735 [0.565-0.878] | **0.020** | 1.5×10^6^ | 0.923 | 0.621 |
| ICAM 1 | 0.499 [0.302-0.700] | 0.969 | N/A | N/A | N/A |

Significant results (p < 0.05) are shown in **bold**.

Abbreviations: AUC, Area under the Curve; CI, Confidence Interval; OR, Odds Ratio; IQR, Interquartile Range; N/A, Not Applicable.

**Table S9. Baseline characteristics and clinical outcomes of immunosuppressed patients excluded from the immunocompetent sensitivity analysis**

| **Characteristics** | **Immunosuppressed patients (N=7)**  **N (%)** |
| --- | --- |
| Age (years), median (range) | 62 (47–91) |
| Sex, Female | 5 (71.4) |
| Charlson Index Median (IQR) | 6 (3–9) |
| High Charlson Index (≥5) | 4 (57.1) |
| Cause of immunosuppresion | |
| - Chemotherapy/radiotherapy in the last 3 months | 4 (57.1) |
| - Chronic systemic corticosteroids | 1 (14.3) |
| - Other immunosuppressive drugs | 2 (28.6) |
| **Infection characteristics** | |
| Peritonitis etiology | |
| - Bowel perforation | 6 (85.7) |
| - Appendicitis | 1 (14.3) |
| Baseline SOFA, median (IQR) | 2 (1.5–2) |
| Baseline Sepsis-3 criteria | |
| - No sepsis | 2 (28.6) |
| - Sepsis | 4 (57.1) |
| - Septic Shock | 1 (14.3) |
| Secondary bacteremia | 0 |
| Positive peritoneal culture | 7 (100.0) |
| Polymicrobial peritoneal culture | 6 (85.7) |
| **Treatment** | |
| Complete source control | 7 (100.0) |
| Time to surgery, days, median (IQR) | 0 (0–1) |
| **Outcomes** | |
| ICU admission | 6 (85.7) |
| ICU length of stay, days, median (IQR) | 4 (3.25–5.50) |
| Severity at follow-up S2 (n=7) | |
| - No sepsis | 2 (28.6) |
| - Sepsis | 4 (57.1) |
| - Septic Shock | 1 (14.3) |
| Hospital length of stay, days, median (IQR) | 20 (14.5–33.5) |
| Overall in-hospital mortality | 1 (14.3) |
| 30-day readmission among survivors (n=6) | 0 (0.0) |

Data are presented as count (percentage) for categorical variables, median (interquartile range, IQR) for continuous variables, and median (range) for age. The denominator for percentages is the immunosuppressed subgroup (n=7) unless otherwise specified. Thirty-day readmission was calculated among survivors discharged alive (n = 6). Overall in-hospital mortality and 30-day mortality were identical in this subgroup.

Abbreviations: ICU, intensive care unit; IQR, interquartile range; SOFA, Sequential Organ Failure Assessment; S2, follow-up timepoint 2.

**Table S10. Baseline immune biomarker levels in immunocompetent peritonitis patients versus healthy controls.**

| **Biomarker**  Median [IQR] | **Controls**  (n=31) | **Immunocompetent peritonitis cohort** (n=35) | **Fold**  **change** | **p-value**  (raw) | **q-value** |
| --- | --- | --- | --- | --- | --- |
| GzmA | 40.69 [33.96–47.42] | 105.88 [77.09–157.26] | 2.60 | **8.17 × 10⁻¹²** | **1.63 × 10⁻¹¹** |
| GzmA activity | 0.00 [0.00– 4.17 × 10⁻⁸] | 4.44 × 10⁻⁸ [0.00–1.24 × 10⁻⁷] | – | **0.006** | **0.006** |
| GzmB | 19.55 [13.44–25.66] | 17.71 [14.78–23.19] | 0.91 | 0.253 | 0.267 |
| TNF-α | 9.45 [6.48–12.42] | 27.94 [20.64–48.76] | 2.96 | **2.70 × 10⁻⁹** | **5.70 × 10⁻⁹** |
| IL-6 | 1.41 [1.01–1.81] | 417.09 [153.88–771.74] | 296.65 | **7.99 × 10⁻¹²** | **6.20 × 10⁻¹¹** |
| IL-8 | 7.76 [5.35–10.17] | 144.46 [75.16–315.93] | 18.61 | **9.79 × 10⁻¹²** | **6.20 × 10⁻¹¹** |
| IL-1β | 5.60 [3.94–7.26] | 15.43 [11.72–19.35] | 2.75 | **1.17 × 10⁻⁷** | **2.03 × 10⁻⁷** |
| IFN-γ | 30.48 [15.87–45.09] | 44.73 [25.57–47.54] | 1.47 | **0.008** | **0.010** |
| IL-10 | 3.82 [1.89–5.75] | 12.11 [8.14–34.62] | 3.17 | **2.50 × 10⁻⁸** | **4.74 × 10⁻⁸** |
| IL-1Rα | 467.13 [339.31–594.95] | 6,810 [3,519–2.43 × 10⁴] | 14.58 | **3.61 × 10⁻¹²** | **6.20 × 10⁻¹¹** |
| CXCL10 | 23.57 [20.50–26.64] | 50.13 [29.45–90.71] | 2.13 | **1.16 × 10⁻⁴** | **1.57 × 10⁻⁴** |
| CXCL9 | 557.24 [557.24–557.24] | 557.24 [551.94–618.93] | 1.00 | 0.116 | 0.130 |
| CCL3 | 327.03 [241.45–412.61] | 483.23 [373.68–636.22] | 1.48 | **1.36 × 10⁻⁴** | **1.72 × 10⁻⁴** |
| IL-17 | 12.88 [12.88–12.88] | 12.88 [7.06–12.88] | 1.00 | 0.558 | 0.558 |
| D-dimer | 6.31 × 10⁵ [3.51×10⁵–9.11×10⁵] | 5.74 × 10⁶ [4.71 × 10⁶–8.26 × 10⁶] | 9.09 | **1.40 × 10⁻¹¹** | **6.64 × 10⁻¹¹** |
| VCAM-1 | 4.66 × 10⁵ [3.80×10⁵–5.51×10⁵] | 1.65 × 10⁶ [1.05 × 10⁶–2.73 × 10⁶] | 3.53 | **6.72 × 10⁻¹¹** | **2.15 × 10⁻¹⁰** |
| ICAM-1 | 5.18 × 10⁵ [3.36×10⁵–6.99×10⁵] | 9.50 × 10⁵ [8.18 × 10⁵–1.40 × 10⁶] | 1.84 | **2.22 × 10⁻⁵** | **3.25 × 10⁻⁵** |
| E-selectin | 2.02 × 10⁴ [1.53×10⁴–2.52×10⁴] | 4.77 × 10⁴ [3.38 × 10⁴–7.87 × 10⁴] | 2.36 | **9.58 × 10⁻¹⁰** | **2.27 × 10⁻⁹** |
| vWF-α2 | 4,710 [3,210–6,200] | 1.54 × 10⁴ [9,160–2.68 × 10⁴] | 3.27 | **6.79 × 10⁻¹¹** | **2.15 × 10⁻¹⁰** |
| Procalcitonin | 38.41 [23.45–53.37] | 952.34 [205.92–1,650] | 24.79 | **1.85 × 10⁻¹⁰** | **5.02 × 10⁻¹⁰** |
| VEGF | 63.22 [51.29–75.15] | 134.94 [84.65–210.40] | 2.13 | **5.09 × 10⁻⁶** | **8.06 × 10⁻⁶** |

Comparisons between immunocompetent peritonitis patients and healthy controls were performed using two-sided Mann–Whitney U tests. Analysis included 35 immunocompetent patients with secondary peritonitis and 31 controls. Fold change was calculated as the ratio of the median value in the immunocompetent peritonitis cohort to the median value in healthy controls. Q-values were calculated using the Holm method for GzmA concentration and GzmA activity, and the Benjamini–Hochberg false discovery rate procedure for biomarkers measured in the multiplex Luminex panel. Significant raw p-values and q-values (p/q < 0.05) are shown in bold.

Abbreviations: IQR, interquartile range.

**Table S11a.** **Baseline immune biomarker levels in immunocompetent peritonitis patients (n=35) stratified by baseline disease severity.**

| **Biomarker**  Median [IQR] | **No sepsis (n=10)** | **Sepsis**  **(n=15)** | **Shock**  **(n=10)** | **Fold change (Shock / No-sepsis)** | **p-value**  **(raw)** | **q-value** |
| --- | --- | --- | --- | --- | --- | --- |
| GzmA | 107.38 [74.02–147.27] | 80.59 [74.50–110.48] | 171.84 [148.41–263.46] | 1.60 | **0.021** | **0.042** |
| GzmA activity | 6.51 × 10⁻⁸  [1.42 × 10⁻⁸–1.09 × 10⁻⁷] | 3.26 × 10⁻⁸ [0–1.09 × 10⁻⁷] | 9.62 × 10⁻⁸  [1.52 × 10⁻⁸–2.12 × 10⁻⁷] | 1.48 | 0.231 | 0.231 |
| GzmB | 17.51 [13.99–20.41] | 17.65 [14.87–20.97] | 19.46 [15.78–26.44] | 1.11 | 0.504 | 0.563 |
| TNF-α | 21.59 [7.57–31.21] | 31.20 [22.21–46.23] | 46.81 [26.34–103.62] | 2.17 | 0.076 | 0.182 |
| IL-6 | 266.81 [104.65–432.70] | 473.10 [171.12–1,677] | 461.04 [370.00–1,756] | 1.73 | 0.202 | 0.310 |
| IL-8 | 68.79 [40.12–136.32] | 147.93 [106.71–315.93] | 309.57 [127.22–912.41] | 4.50 | **0.028** | 0.182 |
| IL-1β | 11.77 [10.10–15.84] | 17.03 [12.26–23.42] | 14.10 [12.22–17.68] | 1.20 | 0.323 | 0.409 |
| IFN-γ | 29.62 [18.09–43.72] | 46.21 [38.35–51.89] | 44.96 [35.50–46.21] | 1.52 | 0.212 | 0.310 |
| IL-10 | 8.14 [3.08–11.86] | 12.30 [9.42–33.92] | 28.31 [13.54–71.55] | 3.48 | 0.058 | 0.182 |
| IL-1Rα | 7,295 [2,028–1.91 × 10⁴] | 7,140 [4,325–2.78 × 10⁴] | 6,810 [4,545–1.76 × 10⁴] | 0.93 | 0.667 | 0.667 |
| CXCL10 | 43.41 [22.34–64.47] | 43.42 [31.52–66.04] | 133.71 [55.79–207.56] | 3.08 | 0.146 | 0.287 |
| CXCL9 | 557.24 [482.53–557.24] | 557.24 [524.80–557.24] | 779.98 [557.24–1,163] | 1.40 | 0.059 | 0.182 |
| CCL3 | 422.67 [290.81–548.76] | 483.23 [374.48–643.23] | 505.00 [442.07–633.08] | 1.19 | 0.483 | 0.563 |
| IL-17 | 12.88 [7.37–12.88] | 12.88 [7.56–12.88] | 9.02 [7.06–12.42] | 0.70 | 0.562 | 0.593 |
| D-dimer | 5.02 × 10⁶  [4.39 × 10⁶–7.93 × 10⁶] | 7.34 × 10⁶  [5.04 × 10⁶–1.23 × 10⁷] | 5.04 × 10⁶  [4.65 × 10⁶–6 × 10⁶] | 1.01 | 0.076 | 0.182 |
| VCAM-1 | 9.55 × 10⁵  [7.22 × 10⁵–1.15 × 10⁶] | 1.5 × 10⁶  [1.09 × 10⁶–2.48 × 10⁶] | 2.68 × 10⁶  [2.33 × 10⁶–3.08 × 10⁶] | 2.81 | **0.002** | **0.046** |
| ICAM-1 | 1.06 × 10⁶  [8.59 × 10⁵–1.25 × 10⁶] | 9.67 × 10⁵  [8.2 × 10⁵–1.92 × 10⁶] | 8.31 × 10⁵  [7.49 × 10⁵–9.41 × 10⁵] | 0.78 | 0.301 | 0.409 |
| E-selectin | 4.51 × 10⁴  [2.84 × 10⁴–7.04 × 10⁴] | 5.43 × 10⁴  [4.2 × 10⁴–8.98 × 10⁴] | 4.5 × 10⁴  [3.38 × 10⁴–7.21 × 10⁴] | 1.00 | 0.200 | 0.310 |
| vWF-α2 | 1.56 × 10⁴  [1.31 × 10⁴–2.21 × 10⁴] | 2.33 × 10⁴  [1.42 × 10⁴–3.03 × 10⁴] | 9,160 [9,160–1.62 × 10⁴] | 0.59 | **0.048** | 0.182 |
| Procalcitonin | 335.55 [66.18–917.28] | 952.34 [335.89–1,526] | 1,510 [920.69–2,028] | 4.50 | **0.045** | 0.182 |
| VEGF | 98.14 [83.76–109.34] | 134.94 [87.53–194.64] | 199.03 [157.40–392.62] | 2.03 | 0.151 | 0.287 |

**Table S11b. Selected post-hoc pairwise severity comparisons in immunocompetent patients**

| **Biomarker** | **Comparison** | **p-value (raw)** | **q-value** |
| --- | --- | --- | --- |
| GzmA | No sepsis vs Sepsis | 0.508 | 0.508 |
|  | No sepsis vs Septic shock | 0.058 | 0.115 |
|  | Sepsis vs Septic shock | 0.006 | **0.018** |
| TNF-alpha | No sepsis vs Septic shock | 0.025 | 0.074 |
| IL-8 | No sepsis vs Sepsis | 0.043 | 0.086 |
|  | No sepsis vs Septic shock | 0.010 | **0.030** |
| IL-10 | No sepsis vs Septic shock | 0.021 | 0.062 |
| CXCL9 | Sepsis vs Septic shock | 0.024 | 0.072 |
| D-dimer | Sepsis vs Septic shock | 0.031 | 0.094 |
| VCAM-1 | No sepsis vs Sepsis | 0.041 | 0.083 |
|  | No sepsis vs Septic shock | 5.21x10^-4^ | **0.002** |
|  | Sepsis vs Septic shock | 0.078 | 0.083 |
| vWF-alpha2 | Sepsis vs Septic shock | 0.014 | **0.041** |
| Procalcitonin | No sepsis vs Septic shock | 0.013 | **0.038** |

Data are presented as medians. Analysis included 35 immunocompetent patients with secondary peritonitis, stratified according to baseline Sepsis-3 severity: No sepsis (n = 10), Sepsis (n = 15), and Septic Shock (n = 10). Global comparisons across baseline severity groups were performed using Kruskal–Wallis test. Fold change was calculated as the ratio of group medians (septic shock/no-sepsis). q-values for GzmA concentration and GzmA activity were adjusted using the Holm method, whereas q-values for the multiplex Luminex panel were adjusted using the Benjamini–Hochberg false discovery rate procedure. Post-hoc pairwise comparisons were assessed using Dunn’s test with Holm correction within each biomarker when appropriate. P-values < 0.05 were considered statistically significant and are highlighted in bold.

Abbreviations: IQR, interquartile range.

**Table S12. Comparison of immune biomarker levels in immunocompetent peritonitis patients (n=35) at baseline stratified by overall mortality**

| **Biomarker/ Subgroup** | **Survivors Median [IQR] (n=23)** | **Non-survivors Median [IQR] (n=12)** | **Fold change** | **p-value** | **q-value** |
| --- | --- | --- | --- | --- | --- |
| GzmA | 99.23 [72.58–-146] | 172 [81.56–-319] | 1.73 | **0.025** | **0.050** |
| GzmA activity | 4.62×10⁻⁸  [2.08×10⁻⁹–-1.24×10⁻⁷] | 1.93×10⁻⁸ [0–-1.24×10⁻⁷] | 0.42 | 0.609 | 0.609 |
| GzmB | 18.55 [15.57–-23.19] | 16.33 [11.43–-20.00] | 0.88 | 0.281 | 0.594 |
| TNF-α | 27.94 [20.71–-54.90] | 30.02 [20.83–-45.63] | 1.07 | 0.848 | 0.948 |
| IL-6 | 378 [144–-516] | 499 [310–-2,354] | 1.32 | 0.259 | 0.594 |
| IL-8 | 112 [68.79–-249] | 198 [130–-373] | 1.77 | 0.231 | 0.594 |
| IL-1β | 15.43 [10.92–-23.42] | 13.89 [12.18–-17.48] | 0.90 | 0.715 | 0.906 |
| IFN-γ | 44.73 [25.57–-51.54] | 44.90 [29.80–-46.21] | 1.00 | 0.714 | 0.906 |
| IL-10 | 11.45 [7.710–-39.80] | 19.48 [9.752–-27.99] | 1.70 | 0.715 | 0.906 |
| IL-1Rα | 6,810 [3,255–-17,482] | 14,147 [4,806–-33,700] | 2.08 | 0.204 | 0.594 |
| CXCL10 | 50.13 [28.22–-79.90] | 50.71 [32.58–-96.65] | 1.01 | 0.958 | 0.986 |
| CXCL9 | 557 [502–-587] | 557 [557–-701] | 1.00 | 0.149 | 0.594 |
| CCL3 | 464 [374–-636] | 505 [392–-626] | 1.09 | 0.986 | 0.986 |
| IL-17 | 12.88 [7.689–-12.88] | 9.025 [3.449–-12.88] | 0.70 | 0.195 | 0.594 |
| D-dimer | 5.04×10⁶  [4.53×10⁶–-7.77×10⁶] | 6.26×10⁶  [5.06×10⁶–-9.93×10⁶] | 1.24 | 0.266 | 0.594 |
| VCAM-1 | 1.18×10⁶  [9.92×10⁵–-2.07×10⁶] | 2.58×10⁶  [1.88×10⁶–-3.23×10⁶] | 2.19 | **0.016** | 0.299 |
| ICAM-1 | 9.57×10⁵  [8.2×10⁵–-1.35×10⁶] | 8.97×10⁵  [7.11×10⁵–-1.39×10⁶] | 0.94 | 0.626 | 0.906 |
| E-selectin | 46,586 [35,320–-75,361] | 51,005 [33,845–-87,641] | 1.09 | 0.554 | 0.906 |
| vWF-α2 | 15,725 [9,160–-27,865] | 14,777 [9,160–-23,076] | 0.94 | 0.562 | 0.906 |
| Procalcitonin | 984 [131–-1,650] | 820 [385–-1,662] | 0.83 | 0.835 | 0.948 |
| VEGF | 107 [80.48–-156] | 195 [159–-289] | 1.83 | **0.036** | 0.337 |

Comparisons between survivors and non-survivors were performed using two-sided Mann–Whitney U tests. Analysis included 35 immunocompetent patients with secondary peritonitis. Data are presented as median [IQR]. Fold change was calculated as the ratio of the median value in non-survivors to that in survivors. Q-values were calculated using the Holm method for GzmA concentration and GzmA activity, and the Benjamini–Hochberg false discovery rate procedure for biomarkers measured in the multiplex Luminex panel. Significant p-values (p < 0.05) and q-values (q < 0.05) are shown in bold.

Abbreviations: IQR, interquartile range

**Table S13. Association of biomarkers with outcomes after exclusion of immunosuppressed patients: univariable logistic regression models.**

| **Outcome** | **Predictor** | **OR** | **95% CI** | **p-value** |
| --- | --- | --- | --- | --- |
| Overall in-hospital mortality  (n=35) | GzmA | 2.82 | 1.10–7.19 | **0.030** |
|  | VCAM-1 | 2.86 | 1.15–7.10 | **0.023** |
|  | VEGF | 1.85 | 0.93–3.69 | 0.080 |
| Persistent sepsis/shock at S2  (n=30) | GzmA | 1.83 | 0.71–4.72 | 0.211 |
|  | IL-8 | 6.02 | 1.53–23.65 | **0.010** |
|  | VCAM-1 | 2.60 | 0.96–7.02 | 0.060 |
|  | vWF-α2 | 1.04 | 0.49–2.18 | 0.919 |
|  | Procalcitonin | 2.96 | 1.38–6.35 | **0.005** |

Univariable logistic regression models were fitted in the immunocompetent peritonitis cohort (n = 35; 12 deaths and 23 survivors) and with available S2 clinical assessment (n = 30; 19 with persistent sepsis/shock). Continuous biomarker variables were log2-transformed; odds ratios represent the change in odds per doubling of the biomarker value. P-values < 0.05 are highlighted in **bold**.

Abbreviations: CI, confidence interval; GzmA, granzyme A; OR, odds ratio; VCAM-1, vascular cell adhesion molecule-1; VEGF, vascular endothelial growth factor, vWF, von Willebrand factor.

**Table S14.** **Discriminatory performance of biomarkers for 30-day in-hospital mortality after exclusion of immunosuppressed patients (n=35, 9 events)**

| **Biomarker** | **AUC [95% CI]** | **p-value** | **Cut-off** | **Sensitivity** | **Specificity** |
| --- | --- | --- | --- | --- | --- |
| SOFA | 0.791 [0.592–0.940] | **0.010** | 4 | 0.889 | 0.615 |
| GzmA | 0.671 [0.436–0.859] | 0.136 | 165.4 | 0.556 | 0.846 |
| GzmA activity | 0.346 [0.177–0.547] | 0.174 | N/A | N/A | N/A |
| GzmB | 0.436 [0.207–0.671] | 0.584 | N/A | N/A | N/A |
| TNF-α | 0.547 [0.325–0.744] | 0.692 | 17.21 | 1.000 | 0.192 |
| IL-6 | 0.654 [0.444–0.838] | 0.180 | 552.1 | 0.556 | 0.769 |
| IL-8 | 0.650 [0.449–0.833] | 0.193 | 139.1 | 0.778 | 0.538 |
| IL-1β | 0.489 [0.263–0.724] | 0.940 | N/A | N/A | N/A |
| IFN-γ | 0.496 [0.276–0.701] | 0.985 | N/A | N/A | N/A |
| IL-10 | 0.543 [0.346–0.735] | 0.720 | 10.18 | 0.778 | 0.423 |
| IL-1Rα | 0.620 [0.368–0.829] | 0.299 | 2.12 × 10⁴ | 0.556 | 0.769 |
| CXCL10 | 0.521 [0.299–0.739] | 0.865 | 75.65 | 0.444 | 0.731 |
| CXCL9 | 0.650 [0.449–0.821] | 0.187 | 526.1 | 1.000 | 0.269 |
| CCL3 | 0.564 [0.333–0.786] | 0.584 | 469.7 | 0.778 | 0.538 |
| IL-17 | 0.421 [0.231–0.641] | 0.488 | N/A | N/A | N/A |
| D-dimer | 0.579 [0.357–0.778] | 0.497 | 5.04 × 10⁶ | 0.889 | 0.423 |
| VCAM-1 | 0.761 [0.556–0.936] | **0.022** | 2.19 × 10⁶ | 0.778 | 0.731 |
| ICAM-1 | 0.485 [0.231–0.727] | 0.910 | N/A | N/A | N/A |
| E-selectin | 0.568 [0.308–0.808] | 0.558 | 8.34 × 10⁴ | 0.444 | 0.923 |
| vWF-α2 | 0.400 [0.188–0.630] | 0.380 | N/A | N/A | N/A |
| Procalcitonin | 0.558 [0.344–0.746] | 0.623 | 107.3 | 1.000 | 0.231 |
| VEGF | 0.598 [0.350–0.842] | 0.396 | 157.1 | 0.778 | 0.654 |

ROC analyses were performed in the immunocompetent peritonitis cohort after excluding immunosuppressed patients (35 patients and 9 events) for 30-day in-hospital mortality. AUCs and 95% confidence intervals were calculated using stratified bootstrap resampling (1,000 iterations). Optimal cut-off values were identified using the Youden index. P-values correspond to univariable logistic regression models using log₂-transformed continuous biomarker values; SOFA was modelled as its raw score. Cut-off, sensitivity and specificity are reported as not applicable (N/A) for biomarkers with AUC < 0.50.

Abbreviations: AUC, area under the receiver operating characteristic curve; CI, confidence interval; GzmA, granzyme A; GzmB, granzyme B; SOFA, Sequential Organ Failure Assessment; VCAM-1, vascular cell adhesion molecule-1; ICAM-1, intercellular adhesion molecule-1; vWF, von Willebrand factor.

**Supplementary Figure S1. Directed acyclic graph (DAG) of hypothesized relationships between GzmA, clinical factors, and mortality in secondary peritonitis.**

**
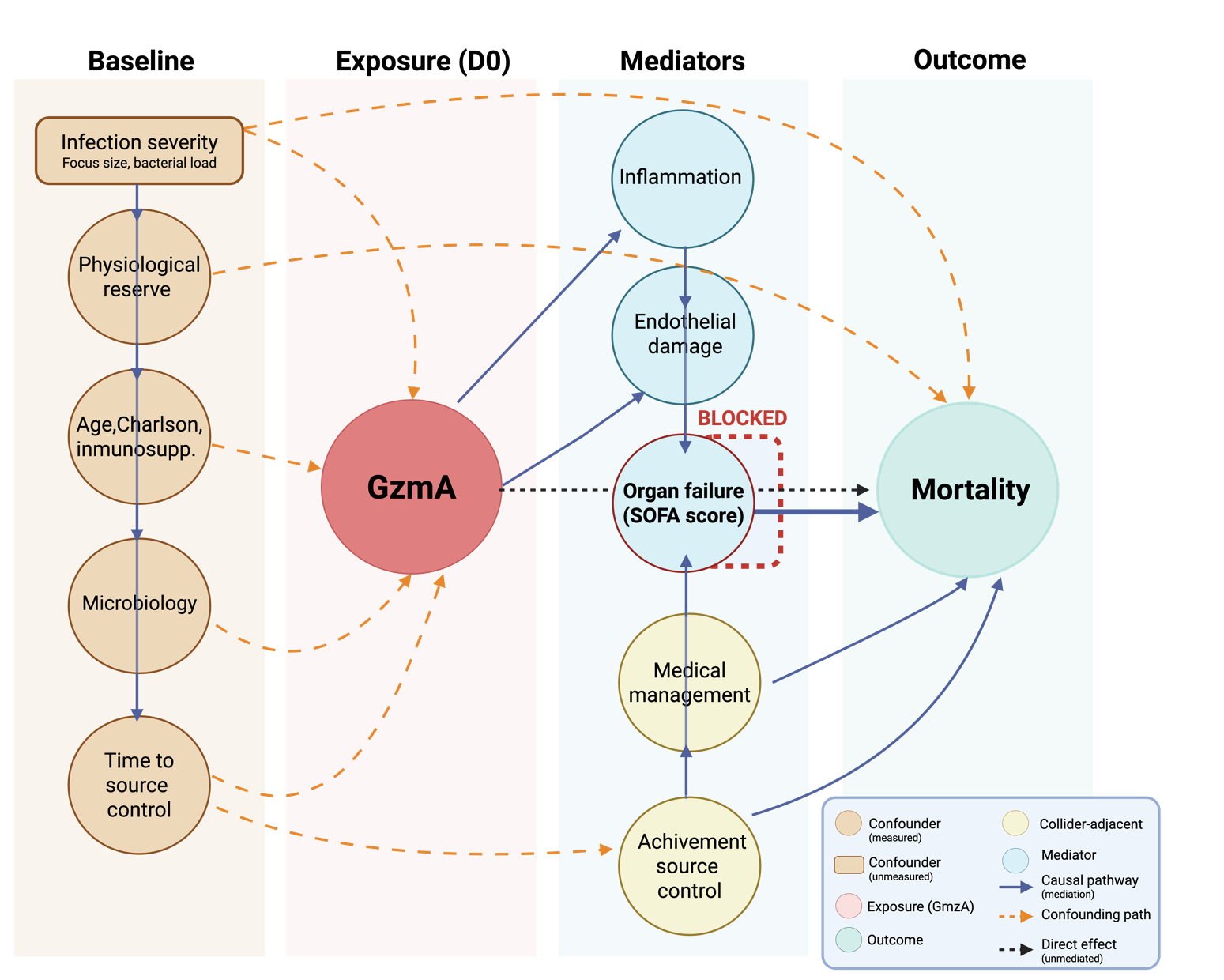
**

**Supplementary Figure S1. Directed acyclic graph (DAG) of hypothesized relationships between GzmA, clinical factors, and mortality in secondary peritonitis.** This DAG is a causal-inference tool used to identify potential confounders and guide covariate selection in regression models. The DAG depicts the assumed causal structure linking baseline factors, circulating GzmA at day 0 (D0), intermediate pathophysiological processes, and mortality. Baseline variables (left panel) act as measured or partially unmeasured confounders (brown nodes). GzmA (red node) represents the exposure of interest. Inflammation and endothelial damage are intermediate mediators (blue nodes) leading to organ failure, captured clinically by the SOFA score. Medical management and achievement of source control are downstream treatment variables that may be influenced by both baseline severity and early organ failure (yellow nodes; collider-adjacent). Mortality is the outcome (green node). Solid blue arrows represent hypothesized causal pathways; orange dashed arrows represent confounding paths; black dashed arrows indicate a possible direct (unmediated) effect of GzmA on mortality. The red “BLOCKED” box highlights SOFA as a mediator on the GzmA–mortality pathway, which was therefore not used for confounder adjustment to avoid mediator overadjustment. This structure motivated adjustment for age and comorbidity in regression models, while avoiding conditioning on post-baseline variables such as SOFA or achievement of source control that could introduce bias.

**Supplementary Figure S2. Baseline levels of additional multiplex biomarkers in patients with peritonitis compared with healthy controls.**

**Supplementary Figure S2. Baseline levels of additional multiplex biomarkers in patients with peritonitis compared with healthy controls.** Scatter plots show circulating levels of IL-1β, IL-8, IL-17, IL-10, IL-1Rα, CXCL9, CXCL10, CCL3, ICAM-1, E-selectin, vWF α2, and procalcitonin in healthy controls (n = 31) and patients with peritonitis (n = 42). Each point represents an individual participant. Horizontal bars indicate median values and error bars the interquartile range (IQR). Comparisons were performed using two-sided Mann–Whitney U tests, and q-values were adjusted using the Benjamini–Hochberg false discovery rate procedure. Significance is displayed according to q-values. All values are shown on a log^₁₀^ scale.

Abbreviations: CCL3, C-C motif chemokine ligand 3; CXCL9, C-X-C motif chemokine ligand 9; CXCL10, C-X-C motif chemokine ligand 10; ICAM-1, intercellular adhesion molecule 1; IQR, interquartile range; IL-1β, interleukin 1 beta; IL-1Rα, interleukin 1 receptor alpha; IL-8, interleukin 8; IL-10, interleukin 10; IL-17, interleukin 17; vWF α2, von Willebrand factor A2. *q < 0.05; ***q < 0.001; ns = non-significant

**Supplementary Figure S3. Exploratory correlation heatmaps of immune biomarkers in the overall peritonitis cohort over time.**

**Supplementary Figure S3. Exploratory correlation heatmaps of immune biomarkers in the overall peritonitis cohort over time.** Triangular heatmaps show Spearman pairwise correlations among circulating immune, endothelial, and coagulation biomarkers measured at baseline (D0, n = 42), early follow-up (S1, n= 39), and later follow-up (S2, n=37) in patients with secondary peritonitis. Only correlations meeting both a magnitude threshold of |ρ| > 0.3 and a BH-adjusted q-values < 0.05 are displayed. Color intensity reflects the strength and direction of the correlation coefficient.

Abbreviations: ρ, Spearman correlation coefficient; BH, Benjamini–Hochberg; D0, baseline; S1, first follow-up; S2, second follow-up.

**Supplementary Figure S4. Heatmap of signed fold-change values across key clinical comparisons in secondary peritonitis.**


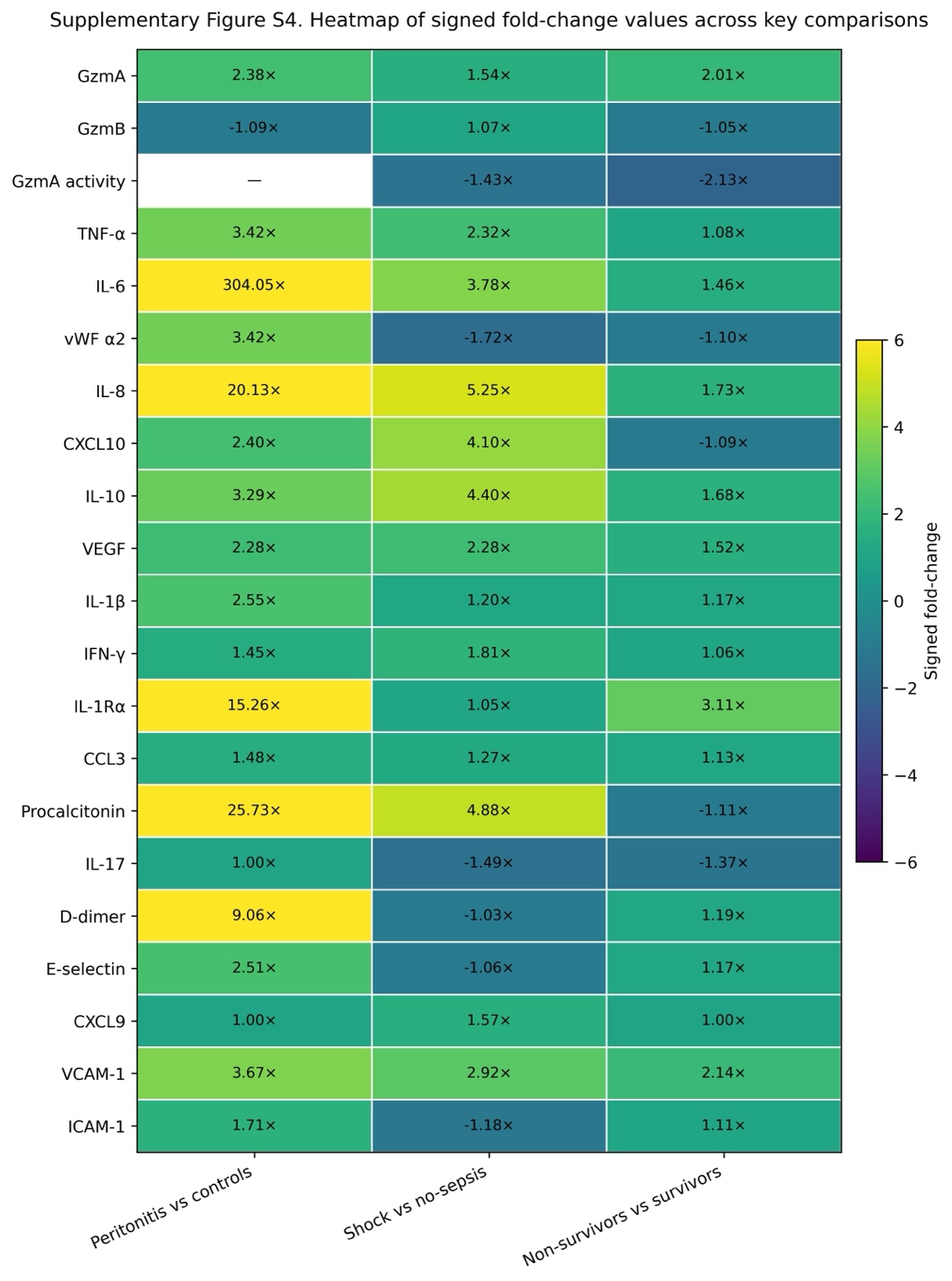


**Supplementary Figure S4. Heatmap of signed fold-change values across key clinical comparisons in secondary peritonitis.** Heatmap showing median biomarker fold-changes for peritonitis patients versus healthy controls (n = 42 vs 31), septic shock versus no sepsis (n = 11 vs 12), and non-survivors versus survivors (n=13 vs 29). Positive values indicate higher median biomarker levels in the first group of each comparison. Fold-changes below 1 are displayed as negative reciprocal signed fold-changes to indicate lower median levels in the first group. Cell labels show the corresponding signed fold-change values. “NA” indicates that fold regulation could not be calculated.

**Supplementary Figure S5. Baseline biomarkers and their association with persistent sepsis/shock at S2 in secondary peritonitis.**

**Figure S5. Baseline biomarkers and their association with persistent sepsis/shock at S2 in secondary peritonitis.** Forest plot of baseline biomarkers in relation to persistent sepsis/shock at S2. The analysis includes patients with available S2 outcome data (n = 37). Squares represent odds ratio (OR) point estimates and horizontal lines indicate 95% confidence intervals (CI); the vertical dashed line represents OR = 1. Biomarker ORs are expressed per doubling of baseline concentration (log₂-transformed), and clinical predictors per per 1-point increase in Charlson index, as detailed in the Methods. Adjusted models in panel include Charlson index.

Abbreviations: CI, confidence interval; GzmA, granzyme A; OR, odds ratio; S2, follow-up (48 h); SOFA, Sequential Organ Failure Assessment; VCAM-1, vascular cell adhesion molecule-1.

**Supplementary Figure S6. Prognostic performance for overall mortality.**

**Supplementary Figure S6. Prognostic performance for overall mortality.** The analysis includes the full peritonitis cohort (n = 42) with 13 overall mortality events. ROC curves evaluating the prognostic performance of the SOFA score and selected baseline immune biomarkers (GzmA, VCAM-1, IL-6) for overall mortality. AUC values are shown. The grey dashed line represents the reference line for no discrimination (AUC = 0.500).

Abbreviations: AUC, area under the curve; CI, confidence interval; GzmA, granzyme A; IL-6, interleukin-6; ROC, receiver operating characteristic; SOFA, Sequential Organ Failure Assessment; VCAM-1, vascular cell adhesion molecule-1.
